# Subgenome evolutionary dynamics in allotetraploid ferns: insights from the gene expression patterns in the allotetraploid species *Phegopteris decursivepinnata* (Thelypteridaceae, Polypodiales)

**DOI:** 10.3389/fpls.2023.1286320

**Published:** 2024-01-09

**Authors:** Natsu Katayama, Takuya Yamamoto, Sakura Aiuchi, Yasuyuki Watano, Tao Fujiwara

**Affiliations:** ^1^ Department of Biological Sciences, Graduate School of Science, The University of Tokyo, Tokyo, Japan; ^2^ Department of Biology, Faculty of Science, Chiba University, Chiba, Japan; ^3^ Department of Biology, Graduate School of Science, Chiba University, Chiba, Japan; ^4^ Center for Molecular Biodiversity Research, National Museum of Nature and Science, Tsukuba, Ibaraki, Japan

**Keywords:** allopolyploid, cytonuclear coordination, expression level dominance, genome evolution, homoeolog expression bias, pteridophyte, subgenome dominance, transcriptome shock

## Abstract

Allopolyploidization often leads to disruptive conflicts among more than two sets of subgenomes, leading to genomic modifications and changes in gene expression. Although the evolutionary trajectories of subgenomes in allopolyploids have been studied intensely in angiosperms, the dynamics of subgenome evolution remain poorly understood in ferns, despite the prevalence of allopolyploidization. In this study, we have focused on an allotetraploid fern—*Phegopteris decursivepinnata*—and its diploid parental species, *P. koreana* (*K*) and *P. taiwaniana* (*T*). Using RNA-seq analyses, we have compared the gene expression profiles for 9,540 genes among parental species, synthetic F_1_ hybrids, and natural allotetraploids. The changes in gene expression patterns were traced from the F_1_ hybrids to the natural allopolyploids. This study has revealed that the expression patterns observed in most genes in the F_1_ hybrids are largely conserved in the allopolyploids; however, there were substantial differences in certain genes between these groups. In the allopolyploids compared with the F_1_ hybrids, the number of genes showing a transgressive pattern in total expression levels was increased. There was a slight reduction in *T*-dominance and a slight increase in *K*-dominance, in terms of expression level dominance. Interestingly, there is no obvious bias toward the *T*- or *K*-subgenomes in the number and expression levels overall, showing the absence of subgenome dominance. These findings demonstrated the impacts of the substantial transcriptome change after hybridization and the moderate modification during allopolyploid establishment on gene expression in ferns and provided important insights into subgenome evolution in polyploid ferns.

## 1 Introduction

Polyploidy, or whole genome duplication, is pervasive in eukaryotes (Van De Peer et al., 2017). It is especially common in plants (Wood et al., 2009), and ancient polyploidization has occurred in most lineages of land plants including mosses (Gao et al., 2022), lycophytes (Xia et al., 2022; Li et al., 2023), ferns (Huang et al., 2020; Pelosi et al., 2022; Chen et al., 2023), gymnosperms (Li et al., 2015), and angiosperms (e.g. Landis et al., 2018). Polyploids are generally categorized into two types: autopolyploids, which arise from simple genome duplication within a single species, and allopolyploids, which result from the merging of entire genomes from more than two species. Allopolyploids possess evolutionary advantages over autopolyploids due to the benefits of both genome duplication and hybridization (Barker et al., 2016). However, the merging of formerly independent genomes may also result in genomic obstacles for allopolyploid species.

Newly formed allopolyploids often experience various deleterious effects due to the rapidity of the genomic change, commonly referred to as “genomic shock” (McClintock, 1984) and “transcriptomic shock” (Hegarty et al., 2006; Buggs et al., 2011). These shocks lead to further genomic modifications and reorganization. The abrupt change in ploidy immediately increases DNA content, resulting in physical changes, such as expanded cell size (Beaulieu et al., 2008) and delayed cell cycle progression (Šímová and Herben, 2012). Genome duplication may also cause dosage imbalances that adversely affect the regulation of dosage-sensitive genes. Beyond the effects of simple genome duplication, allopolyploids may also suffer from the effect of hybridization—a disruptive conflict between the two genomes that have evolved independently but are now merged into one genome. This conflict ranges from negative epistatic interactions (Orr, 1996) and regulatory interference between genes (Kaltenegger and Ober, 2015) to alterations in the DNA methylation pattern (Song and Chen, 2015; Rigal et al., 2016) and changes in gene expression (Yoo et al., 2014). Polyploids must mitigate the adverse effects of polyploidization and hybridization to enable their survival and long-term success (Hegarty and Hiscock, 2008). The necessity for this can trigger subsequent transcriptomic and genomic reorganizations. These reorganizations encompass modifications of gene expression, neo- and sub-functionalization (Lynch and Conery, 2000; Birchler and Yang, 2022), or loss of the duplicated genes (De Smet et al., 2013), and genome shrinkage (Wang et al., 2021), ultimately resulting in complete diploidization of the duplicated genomes (Mandáková and Lysak, 2018; Li et al., 2021).

The aforementioned genomic changes usually initially start with changes in gene expression in response to genomic duplication and hybridization (Hegarty et al., 2006; Buggs et al., 2011; Yoo et al., 2014), and the patterns may vary among polyploids derived from phylogenetically distinct groups. There are two major phenomena that have been broadly reported across taxa (Grover et al., 2012). The first is “expression level dominance (ELD),” which is the phenomenon where the total expression level of a pair of homoeologous genes is similar to that exhibited by only one of the two diploid parents (Rapp et al., 2009; Flagel and Wendel, 2010; Bardil et al., 2011; Sigel et al., 2019; Chen et al., 2021). The second is “homoeolog expression bias (HEB),” which is the phenomenon where two homoeologous genes are expressed unequally compared to prior expectations based on the progenitor diploid expression levels (Chaudhary et al., 2009; Koh et al., 2010; Edger et al., 2017; Sigel et al., 2019; Chen et al., 2021; Vasudevan et al., 2023). Among the studies focusing on angiosperms, while some studies reported that ELD and/or HEB occur randomly on the homoeologous genes from both parental subgenomes (e.g. Boatwright et al., 2018; Boatwright et al., 2021; Burns et al., 2021), the other studies revealed that ELD and/or HEB tend to preferentially biased towards the subgenome from one parental species (e.g. Chaudhary et al., 2009; Koh et al., 2010; Edger et al., 2017; Chen et al., 2021; Vasudevan et al., 2023). The latter phenomenon, known as “subgenome dominance” (Bird et al., 2018; Cheng et al., 2018), is expected to accelerate genome shrinkage by causing a more pronounced fractionation of the recessive subgenome due to relaxed selective constraints. In contrast, gymnosperms, characterized by their highly stable and large genomes, show no subgenome dominance in the cases examined to date (Wu et al., 2021). Consequently, patterns of gene expression that change in subgenomes following polyploidization may differ across several aspects, including the scale and the direction, among different land plant lineages that exhibit the distinct consequences of genomic evolution.

Ferns exhibit the highest frequencies of polyploidization and hybridization among vascular plants (Whitney et al., 2010), with more than 30% of speciation events in ferns estimated to involve polyploidy (Wood et al., 2009). Not only does the frequent formation of neo-polyploids contribute to the statistics, but there is also increasing evidence for paleo-polyploid origins in the majority of lineages (Huang et al., 2020; Pelosi et al., 2022; Chen et al., 2023). However, the genomic consequences of polyploid evolution in ferns appear to differ from those in angiosperms (Barker, 2013). In angiosperms, most lineages conserve small genome sizes and chromosome numbers despite frequent WGDs in the evolutionary history, suggesting fast diploidization including dysploidal reduction and genome downsizing in this group (Wang et al., 2021). In contrast to angiosperms, in the ferns, the genome is generally highly stable in terms of chromosome number and genome size (Clark et al., 2016). The evolutionary trajectory of the polyploid fern genome is thought to be characterized by slow reductions in chromosome numbers and gradual genome downsizing (Barker, 2013; Haufler, 2014). This is exemplified by the positive correlation between chromosome number and genome size observed in whole ferns (Clark et al., 2016; Fujiwara et al., 2023). These differences in the macroevolutionary patterns of genome evolution between angiosperms and ferns indicate the potential variations in the short-term consequences of gene expression changes in their subgenomes following allopolyploid formation. However, our current understanding of ferns from this perspective remains limited, as gene expression patterns have been examined for only one allotetraploid fern to date (Sigel et al., 2019). Sigel et al. demonstrated that the allotetraploid fern, *Polypodium hesperium* Maxon, exhibits a strong subgenome dominance, with the number of genes showing ELD and HEB being extremely biased toward one parental subgenome. However, it remains unclear whether the reported pattern is universal across all allopolyploid ferns. Additionally, as the study explored the transcriptome-wide patterns only through comparisons of the parental species and allopolyploids, it is unknown whether the observed pattern was primarily driven by the effects of hybridization or polyploidization (Hegarty et al., 2006; Edger et al., 2017; Duan et al., 2023). To achieve a comprehensive understanding of gene expression changes during allopolyploidization in ferns, more case studies involving diverse allopolyploid fern species will be required. Furthermore, comparing expression patterns between homoploid hybrids and allopolyploids of the same parental species pair could help to distinguish the effects of interspecific hybridization and those of the events that follow polyploidization.

To explore gene expression pattern changes in response to hybridization and polyploidization in ferns, we have focused on *Phegopteris decursivepinnata* (H. C. Hall) Fée. This species, which is tetraploid (2*n* = 4*x* = 120), is mainly distributed across East Asia. While this fern was previously presumed to be an autopolyploid of a diploid *P. decursivepinnata* strain (Nakato et al., 2012; Kawakami et al., 2019), a recent phylogenetic study revealed that the species is actually an allotetraploid resulting from hybridization between *P. koreana* B. Y. Sun & C. H. Kim (previously treated as diploid *P. decursivepinnata*) (2*n* = 2*x* = 60) and *P. taiwaniana* T. Fujiw., Ogiso & Seriz. (2*n* = 2*x* = 60) (Fujiwara et al., 2021). Although the two parental species occupy different ecological niches, cool temperate forests and warm temperate to subtropical forests, they are in close geographical proximity to each other in the Japanese archipelago. Considering that both parental species still exist and are geographically close to the allopolyploid species, it is speculated that the two parental species hybridized, producing the allotetraploid relatively recently. Therefore, it provides an opportunity to examine the initial gene expression changes that occur in allopolyploid speciation. This species is also relatively easy to cultivate in a laboratory setting, as evidenced by previous studies on the mating system of gametophytes and artificial manipulation of ploidy (Masuyama, 1979; Masuyama, 1986; Kawakami et al., 2019; Nakato and Masuyama, 2021). Artificial F_1_ hybrids can also be created between the parental species, facilitating comparisons with natural allopolyploids. This study system thus offers the potential to discern the effects of hybridization and polyploidization on gene expression patterns in allopolyploid species.

In this study, we have conducted a comparison of the transcriptome profiles to investigate gene expression changes between the F_1_ hybrids and natural allopolyploids. Because there is no available published genome for *Phegopteris*, we construct *de-novo* reference transcriptomes using only single-copy genes with a one-to-one correspondence between the parental species and focused on co-expressed genes among the parental species, F_1_ hybrids, and allopolyploids. Using the dataset, we aimed to clarify the evolutionary implications of hybridization and polyploidization on gene expression changes. In particular, the phenomena of ELD and HEB were assessed to explore the evolutionary trends that occur during fern subgenome evolution.

## 2 Materials and methods

### 2.1 Plant materials

To create F_1_ hybrids, gametophytes were cultivated from the parental diploid species, *Phegopteris koreana* and *P. taiwaniana*, on agar media in petri dishes. The spores sown were collected from two samples of *P. koreana* and two samples of *P. taiwaniana* (**Supplementary Table S1**). The culture conditions were primarily based on those outlined by Watano and Masuyama (1991). From each of the two species, one gametophyte (total of two) was transplanted into the same well of a 12-well petri dish. Sporophytic outcrossing or gametophytic selfing (Haufler et al., 2016) was then promoted with the addition of two to three drops of sterilized water to each well once a week. The resulting sporophytes were transferred into pots individually and cultivated in the experimental field at the Nishi-chiba campus of Chiba University. To evaluate the hybridity of the produced sporophytes, Sanger sequencing was conducted for the plastid DNA marker, *rbcL*, and the single-copy nuclear gene, *PgiC*. Subsequently, phylogenetic analyses were performed for each gene by incorporating the sequences into the previous sequence matrix used by Fujiwara et al. (2021). The experimental procedures, from PCR to phylogenetic analysis, followed the methods described by Fujiwara et al. (2021). As a result, three F_1_ hybrid individuals with *P. koreana* as a maternal parent (referred as to F_1K_) and two F_1_ hybrid individuals with *P. taiwaniana* as a maternal parent (referred as to F_1T_) were obtained. The maternal diploid ancestor of the allotetraploid *P. decursivepinnata* has been proven to be *P. koreana* (Fujiwara et al., 2021), and thus the F_1K_ hybrids were verified to be the products of crossing in the same direction as *P. decursivepinnata*, while the F_1T_ hybrids were in the opposite direction. The sporophytes genetically identified as F_1_ hybrids were used for subsequent analyses (**Supplementary T** **able S2**).

Sporophytes of the parental diploid species, *Phegopteris koreana* and *P. taiwaniana*, were also produced by artificial fertilization of the gametophytes. This was necessary as the parental sporophytes of the F_1_ hybrids (**Supplementary Table S1**) were not cultured. Two gametophytes of the same species were transplanted into the same well of a 12-well petri dish, and sporophytic selfing or gametophytic selfing was promoted as previously described. Genotypes of the resulting sporophytes were determined using the same methods as described for F_1_ hybrid identification. Ultimately, three *P. koreana* and three *P. taiwaniana* sporophytes were selected for subsequent analyses (**Supplementary Table S2**).

For the allotetraploid species, *Phegopteris decursivepinnata*, three sporophyte samples were collected (**Supplementary Table S1**), and then cultivated in an experimental field alongside those of the F_1_ hybrids and parental species, for the period from April 2021 to April 2022 prior to RNA extraction.

### 2.2 RNA extraction

Approximately 2 × 2 cm squares of the unfolding young leaves were collected in April 2021 from three *Phegopteris koreana*, three *P. taiwaniana*, and three F_1K_ individuals, and in April to May 2022 from two F_1T_ and three *P. decursivepinnata* individuals. Additionally, individuals representing each parental species (K1 and T1) were resampled in April 2022 to assess the reproducibility of results across the two years (**Supplementary Ta** **ble S2**). The collected leaf samples were rapidly frozen in liquid nitrogen and subsequently crushed using zirconia beads. Total RNA was extracted using the PureLink™ Plant RNA Reagent (ThermoFisher Scientific). The RNA-seq libraries were constructed using NEBNext^®^ Poly(A) mRNA Magnetic Isolation Module (for PolyA selection) and NEBNext^®^ Ultra™ II Directional RNA Library Prep Kit according to the manufacturer’s instructions. Illumina 150 bp paired-end sequencing was performed in NovaSeq6000. The raw read data was deposited in the DDBJ Sequence Read Archive under the accession numbers (DRR498230–DRR498244).

### 2.3 *De novo* assembly and ortholog inference among *P. koreana*, *P. taiwaniana*, their F_1_ hybrids, and the allotetraploids

For all RNA-seq reads, adapter sequences and low-quality bases (Q < 20) were trimmed using Trimmomatic v.0.39 (Bolger et al., 2014). After trimming, the filtered reads from biological replicates of each species (**Supplementary T** **able S2**) were assembled using Trinity v.2.9.1 (Grabherr et al., 2011). To remove isoform redundancy in the Trinity assembly outputs, the highest expressed isoforms within genes estimated by RSEM (Li and Dewey, 2011) were selected using the “filter_low_expr_transcripts.pl” provided in Trinity v.2.9.1. TransDecoder v.5.5.0 was used to recognize the ORFs, and only the longest ORF per transcript was retained. The obtained sequences were annotated with TAIR IDs based on the best BLASTP hit with a cutoff E-value of 10^–5^ against the *Arabidopsis* protein sequence database (Araport11_pep_20220914_representative_gene_model). Organelle-encoded genes were removed from subsequent analyses. After the above filtering steps, orthogroups were constructed among parental species, F_1K_, F_1T,_ and *Phegopteris decursivepinnata* using OrthoFinder v.2.5.2 (Emms and Kelly, 2019). The venn diagrams showing the numbers of coexpressed orthologs within orthogroups were drawn using the venn package v1.11 in R software (R Core Team, 2022). The orthogroups containing a single gene each in parental species were used as reference gene sets (**Supplementary Table S3**).

### 2.4 Differential expression analysis among species

For the differentially expressed gene (DEG) analyses of the homoeologs in the F_1_ hybrids and the allotetraploids, the reads of the parental species were mapped to their own reference gene sets and the reads from the F_1_ hybrids and the allotetraploids were then mapped to the reference genes in both *P. koreana* and *P. taiwaniana* using bowtie2 v.2.4.5 (Langmead and Salzberg, 2012). Considering that the F_1_ hybrids and the allotetraploids retain homoeologs from both parental species, the output bam files from bowtie2 were used as input for the file for EAGLE-RC (Kuo T. et al., 2018), a tool that identifies the parental origin of the reads from the F_1_ hybrids and the allotetraploids. The number of mapped reads was counted using eXpress v.1.5.1 (Roberts and Pachter, 2013). The edgeR package v.3.40.2 (Robinson et al., 2010) in R, was used to determine the read count data for the replicates and this was normalized using the trimmed mean of *M* values (TMM) methods, and differential expression analyses were performed between parental species, between the F_1_ hybrids and each parent, and between the allotetraploids and each parent. In the present study, the DEGs were identified using Fisher’s exact test, with the following criteria: |log_2_(fold change (FC))| >1 and false discovery rate (FDR) < 0.05. To evaluate the consistency of the trends of the expression patterns of the entire genomes/subgenomes, we conducted the three patterns of transcripts per million (TPM) filtering, TPM > 0.0, TPM > 0.5, and TPM > 1.0, in all five groups.

### 2.5 Analysis of expression level dominance

**Figure 1:**
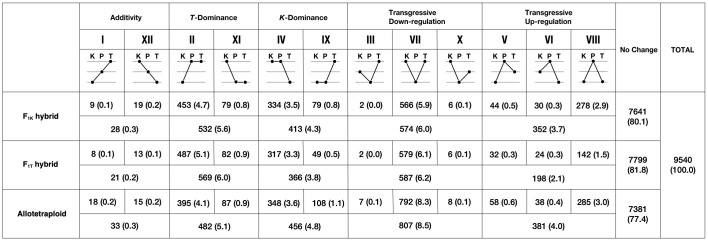
Twelve categories for differential expression states in the F*_1_* hybrids (F_1K_ and F_1T_) and the allotetraploids, *Phegopteris decursivepinnata* relative to the diploid parents. Roman numerals indicate categories as described by Rapp et al. (2009). Numbers show the number of genes assigned to each category and the rates are shown in parentheses. Schematic figures show the gene expression levels relative to the parents (K: *P. koreana*, P: F_1K_, F_1T_, or *P. decursivepinnata*, and T: *P. taiwaniana*).

Genes identified as DEGs in the F_1_ hybrids and the allotetraploids relative to their diploid parents were classified into 12 expression-level categories (**Figure 1**), using the differential expression classification outlined by Rapp et al. (2009). These categories include the following: additivity (I and XII), *Phegopteris taiwaniana* ELD (*T*-dominance; II and XI), *P. koreana* ELD (*K*-dominance; IV and IX), transgressive expression lower than either parent (Transgressive Down-regulation; III, VII, and X), and transgressive expression higher than either parent (Transgressive Up-regulation; V, VI, and VIII). Genes that did not show significant differential expression levels were categorized as “No Change.” When comparing the F_1_ hybrids to the allotetraploids, the 12 categories were grouped into five broader categories (*K*-dominance, *T*-dominance, Additivity, Transgressive Up-regulation, and Transgressive Down-regulation). The changes among the five differential expression categories from the F_1_ hybrids to the allotetraploids were visualized using the qgraph package (v.1.9.5) in R.

### 2.6 Detection of homoeolog expression bias

Using the edgeR package in R, the expression level ratio, log_2_(FC), of the orthologous genes from *Phegopteris taiwaniana* and *P. koreana* was calculated. This calculation involved comparing the mean count-per-million (CPM) of the alleles from *P. taiwaniana* (Pt) replicates with the mean CPM of those from the *P. koreana* replicates (Pk), as follows: log_2_(Pt__ortholog_/Pk__ortholog_). The same method was used to determine the expression level ratios of the parental alleles in the F_1_ hybrids, as follows: log_2_(F_1_T-homoeolog_/F_1_K-homoeolog_), and those of the homoeologs in the allotetraploid *P. decursivepinnata* (Pd): log_2_(Pd__T-homoeolog_/Pd__K-homoeolog_). Subsequently, the distribution of the log_2_(FC) was compared among the parental species, the F_1_ hybrids, and the allotetraploids.

The expression bias of the homoeologs in the F_1_ hybrids and the allotetraploids was analyzed using the existing difference in gene expression levels between the parental species. To achieve this, the ratio of the mean CPM of the orthologous genes in the parental species and the ratio of the mean CPM of homoeologous genes in each of the F_1_ hybrids or the allotetraploids was compared using Fisher’s exact test. The ratio of the log_2_ (FC) for the orthologous genes in the parental species and that of the homoeologs in the F_1_ hybrids was then determined as follows: log_2_((F_1_T-homoeolog_/F_1_K-homoeolog_)/(Pt__ortholog_/Pk__ortholog_)). Similar comparisons were conducted between the parental species and the allotetraploids, as follows: log_2_((Pd__T-homoeolog_/Pd__K-homoeolog_)/(Pt__ortholog_/Pk__ortholog_)). The ratio of log_2_(FC) was referred to as the “magnitude” of the bias. The HEB was detected for each of the homoeologous gene pairs using the following criteria: P-value <0.05 and FDR <0.05 with the Fisher’s exact test, and the absolute value of the “magnitude” >1. The homoeologous gene pairs with a “magnitude” >1 and <−1 are considered as HEB toward *P. taiwaniana* (*T*-bias) and *P. koreana* (*K*-bias), respectively. The relationship of the gene sets exhibiting the HEB between the F_1_ hybrids and the allotetraploids are shown using Circular layout graphs using the qgraph packages in R. The plots that show the relationship between the log_2_(FC) values of parental orthologs and those of homoeologs and the histograms showing the difference in the expression bias between the F_1_ hybrids or the allotetraploids and the parents were generated using the ggplot package in R.

## 3 Results

### 3.1 Artificial F*_1_* hybrid production

The artificial crossing experiment successfully produced several sporophyte individuals. The nuclear *PgiC* tree confirmed that five of these were interspecific F_1_ hybrids between *Phegopteris koreana* and *P. taiwaniana* (**Supplementary Figure S1**). The plastid *rbcL* tree showed that there were three and two hybrids with *P. koreana* and *P. taiwaniana* as their mother, respectively, given that maternal inheritance of organelle genomes in ferns (reviewed in Kuo L.-Y. et al., 2018) (**Supplementary Figure S1**; **Supplementary Table S2**).

### 3.2 Transcriptome profiles and inference of parental orthologous pairs

Approximately, 45–72 millions of 150 bp pair-end raw reads were sequenced in each studied sample. After filtering the RNA-seq reads for quality, the initial transcripts were assembled and filtered to 26,663–31,796 nuclear genes containing complete or partial ORFs across five groups containing three species and reciprocal F_1_ hybrids of the parental species (**Supplementary Table S3**). The ortholog identification using these filtered genes yielded 31,936 orthogroups among the five groups. Of these, 12,380 genes that were detected in both parental species and in which no gene duplication was identified for each parental species, were retained as mapping references for subsequent gene expression analysis (**Supplementary F** **igure S2**). To reveal the effects of the low-expressed genes, we performed three levels of TPM filtering for all five groups, and 12,380 orthologous genes were retained with TPM > 0, 9,540 with TPM > 0.5, and 9,003 with TPM > 1.0 (**Supplementary Table S3**). MDS plot of the expression matrix (**Supplementary Figure S3**) showed that the replicates of each parental species, F_1K_ hybrids, F_1T_ hybrids, and allotetraploids, *Phegoteris decursivepinnnata* are closely clustered together, suggesting that the expression pattern is highly consistent among the replicate samples of each group. The samples from each parental species include those collected in different years (2021 and 2022), but all are closely clustered, and there is no apparent influence due to the difference in years. Furthermore, the clustering result is congruent among the datasets with different TPM filtering. In the comparison of expression levels across species, genes showing stable expression should be used because genes that respond to transient environmental changes should be excluded from the analyses. Therefore, we mainly showed the results obtained from the dataset with TPM > 0.5 for the subsequent analysis.

### 3.3 Expression level dominance in the F_1_ hybrids and the allotetraploid *P. decursivepinnata*

To identify additivity, transgressive expression, and ELD in the F_1_ hybrids and the allotetraploids, in comparison to the expression levels in the parental species, the 9,540 orthologous genes were classified into 12 categories, following the categorization system described by Rapp et al. (2009) (**Figure 1**). The analysis revealed that most genes from the F_1_ hybrids and the allotetraploids did not display significant differential expression levels when compared to the orthologs of their parents (denoted as “No Change” in **Figure 1**; 80.1% in the F_1K_ hybrids, 81.8% in the F_1T_ hybrids, and 77.4% in the allotetraploids), with a lesser proportion being observed in the allotetraploids (2.7% and 4.4% decreases compared to the F_1_ hybrids). Few differences were found in the count for “No Change” genes between the hybrids with *Phegopteris koreana* (F_1K_) and *P. taiwaniana* (F_1T_) as their mothers, respectively. For “Additivity,” the category I and XII showed one of the lowest proportions among the all of categories in the F_1T_ hybrids, the F_1K_ hybrids, and the allotetraploids, and the proportion of the categorized genes was almost consistent between the F_1_ hybrids and the allotetraploids.

For transgressive expression, a substantial number of genes were found to display lower and higher expression levels when compared to both parents (Transgressive Down- and Up-regulation) in the F_1_ hybrids and the allotetraploids. The number of genes in “Transgressive Down-regulation” always outnumbers the number in “Transgressive Up-regulation” across both the F_1_ hybrids and the allotetraploids. Interestingly, the number of genes exhibiting transgressive expression was increased in the allotetraploids, and also the difference in the number of genes between “Transgressive Down-regulation” and “Transgressive Up-regulation” became more pronounced in the allotetraploids: from 574 in the F_1K_ hybrids and 587 in the F_1T_ hybrids to 807 genes in the allotetraploids across “Transgressive Down-regulation” categories (III, VII and X), and from 352 in the F_1K_ hybrids and 198 in the F_1T_ hybrids to 381 genes in the allotetraploids across “Transgressive Up-regulation” categories (V, VIII, and VI).

The analyses have also revealed significant trends in ELD. There were significantly more instances where the F_1_ hybrids and the allotetraploids exhibited the same expression level as the parent with higher expression, regardless of either “*T-*dominance” or “*K-*dominance” categories. For instance, for the category “*T*-dominance” in the F_1K_ hybrids, there were 453 instances where *Phegoteirs taiwaniana* showed higher expression than *P. koreana* (category II), compared to only 79 instances where the opposite was observed (category XI). A similar trend was observed in the F_1T_ hybrids and the allotetraploids (**Figure 1**). In addition, while a higher number of the genes exhibited “*T-*dominance” when compared to “*K-*dominance” in both the F_1K_ and F_1T_ hybrids, the allotetraploids showed a similar proportion of the genes exhibiting “*T-*dominance” and “*K*-dominance”. In the allotetraploids, 395 and 87 genes in categories II and XI, respectively, exhibited “*T-*dominance”, and 348 and 108 genes in categories IV and IX, respectively, exhibited “*K-*dominance”.

**Figure 2:**
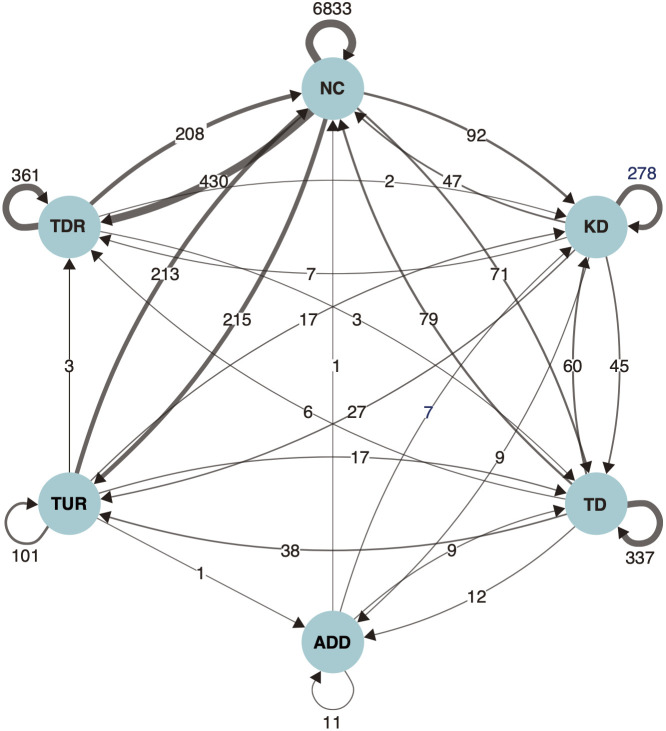
Diagram showing categorical changes in the differential expression of genes from the F_1K_ hybrids to the allotetraploids, *Phegopteris decursivepinnata*.NC, KD, TD, ADD, TUR, and TDR are the abbreviations of “No Change”, “*K*-dominance”, “*T*-dominance”, “Additivity”, “Transgressive Up-Regulation”, and “Transgressive Down-Regulation” respectively. Numbers with arrows indicate the number of genes whose categories changed from the F_1K_ hybrids to the allotetraploids.

The changes in the expression level categories for each gene from the F_1K_ hybrids to the allotetraploids were then examined (**Figure 2**). The F_1K_ hybrids are the product of crossing in the same direction as the natural allotetraploids. Most genes that were categorized as “No Change” (89.4%), “*T-*dominance” (63.3%), and “*K-*dominance” (67.3%) in the F_1_ hybrids also belonged to the same categories in the allotetraploids. The transition between categories occurred the most frequently between “No Change” and “Transgressive-Up-regulation” or “Transgressive Down-regulation” (208–403 genes). Especially, the transition from “No Change” to “Transgressive Down-regulation” detected almost twice as many cases (403 genes) compared to other transitions between “No Change” and transgressive expressions (208–215 genes). In the genes showing ELD, the number of genes that transitioned between “No Change” and “*T*-dominance” are almost the same in both directions (71 and 79 genes), while the number of genes that transitioned from “No-Change” to “*K*-dominance” (92 genes) was almost double that of the other direction (47 genes).

### 3.4 Relative homoeolog contributions and expression biases in the F_1_ hybrids and the allotetraploid *P. decursivepinnata*

**Figure 3:**
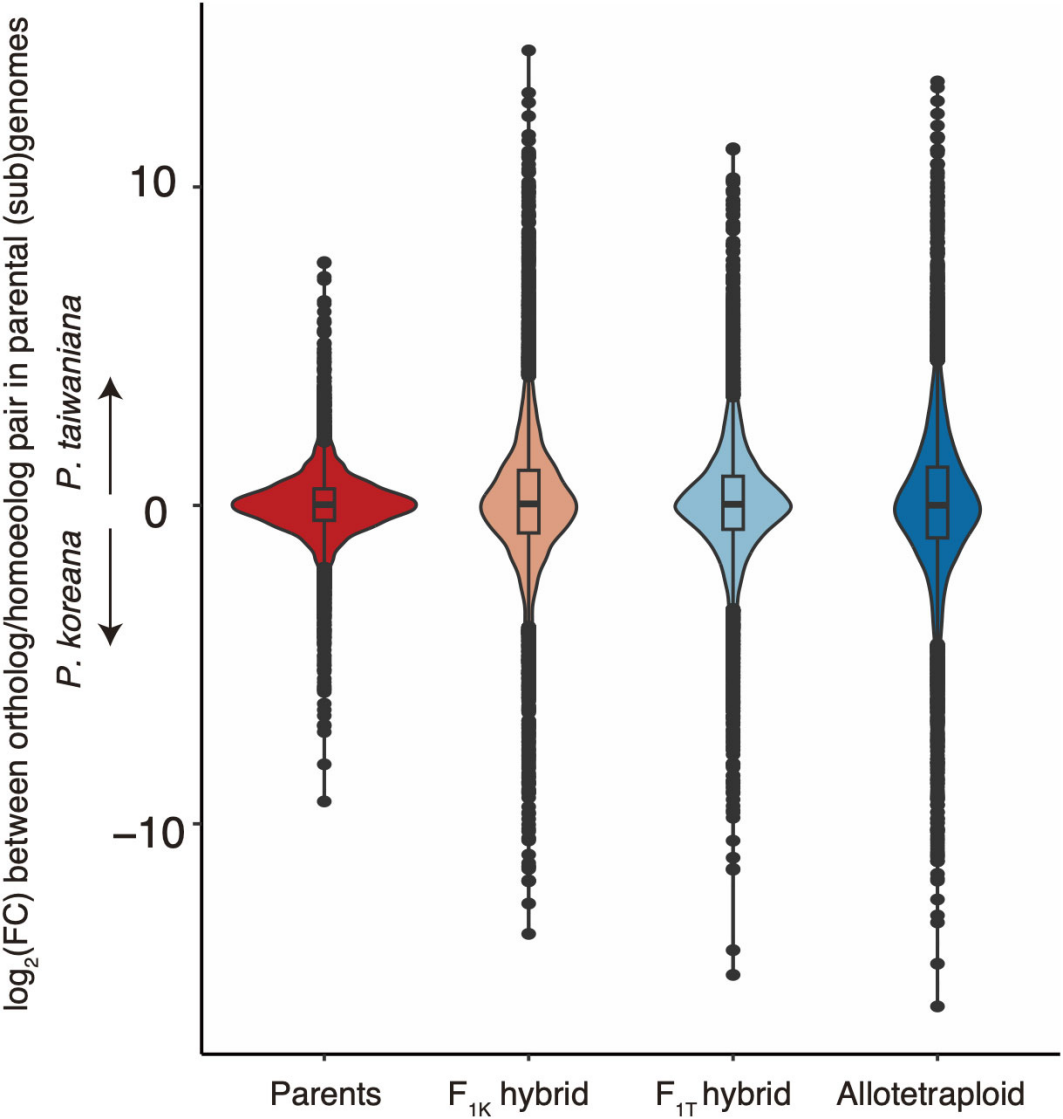
Patterns in the expression level ratio between homoeologs derived from *Phegopteris koreana* and *P. taiwaniana* (sub)genomes. Violin plots showing the log_2_(fold change (FC)) between the orthologous genes of the parental species (*P. koreana* and *P. taiwaniana*), the parental alleles of the F_1K_ and F_1T_ hybrids, and the homoeologous genes of the allotetraploids, *P. decursivepinnata*. The center line indicates the median and the box limits represent the interquartile range. The whiskers represent the largest and smallest values within 1.5 times the interquartile range above and below the 75th and 25th percentiles, respectively.

To test the subgenome dominance of *Phegopteris koreana* or *P. taiwaniana* in the F_1_ hybrids and the allotetraploids, the relative homoeolog contributions and distributions of the expression level ratios for all 9,540 orthologous gene pairs were examined (**Figure 3**). Although significant deviations from the mean of 0 were detected in the parental pairs, the F_1K_ hybrids, and the allotetraploids, an apparent expression bias toward either subgenome was not observed in all cases, as the mean and median values were close to 0 (**Table 1**; **Figure 3**). However, the kurtosis of the distribution was highest in the parental species, and lowest in the allotetraploids. This suggests that the genes with biased expression increased in number after the acquisition of hybridization and polyploidization.

**Table 1:** Statistics of the distribution of log_2_(fold change (FC)) between orthologous or homologous gene pairs.

	mean	median	kurtosis	P value (FDR) for *T* test
**Parents**	0.04	0.02	6.45	<0.001 (<0.001)
**F_1K_ hybrid**	0.14	0.05	4.72	<0.001 (<0.001)
**F_1T_ hybrid**	0.10	0.04	5.17	<0.001 (<0.001)
**Allotetraploid**	0.10	0.00	4.26	<0.001 (<0.001)

**Figure 4:**
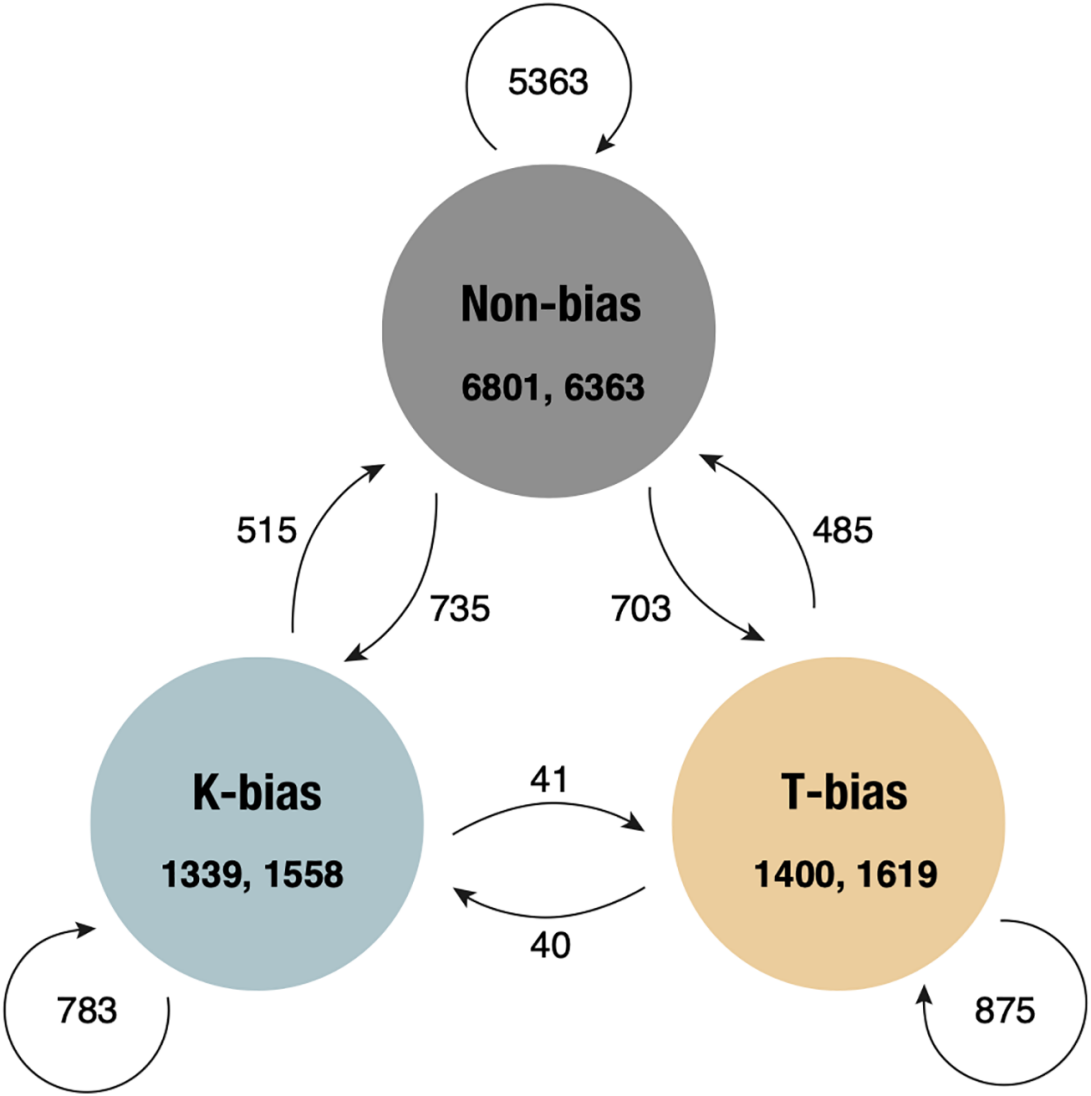
Categorical changes in homoeolog expression bias genes between the F_1K_ hybrids and the allotetraploids, *Phegopteris decursivepinnata*. Numbers with arrows indicate the number of genes that show changes in homoeolog expression bias between the F_1K_ and the allotetraploids. The numbers in each category show the number of genes in the F_1K_ hybrids and the allotetraploids on the left and right, respectively.

To understand the influence of the expression bias of the homoeologous gene pairs on gene expression, while considering the pre-existing differences in the parental species, the expression level ratios between the homoeologs in the F_1_ hybrids and the allotetraploids were compared with those between the parental species. Among the 9,540 orthologous gene pairs, in both the F_1_ hybrids and the allotetraploids, approximately 1,000–1,600 pairs exhibited different expression level ratios when compared with the parental ortholog pairs, a phenomenon referred to as HEB (**Table 2**). Notably, there is no substantial difference between the proportions of *T*- or *K*-biased gene pairs observed in both the F_1_ hybrids and the allotetraploids (1,400 “*T*-bias” vs 1,339 “*K*-bias” in the F_1K_ hybrids, and 1,020 vs 991 in the F_1T_ hybrids, 1,619 vs 1,558 in the allotetraploids). Of the gene pairs showing HEB, 60.5% (1658 gene pairs) were shared between the F_1K_ hybrids and the allotetraploids (**Figure 4**). The majority of the genes in the other 40% varied between the F_1K_ hybrids and the allotetraploids by transitioning to or from the “Non-bias” category, while a limited number of gene pairs, only 40 or 41 gene pairs, showed a direct switch between the “*T*-bias” and “*K*-bias” categories (**Figure 4**). More gene pairs altered their expression ratios from “Non-bias” in the F_1K_ hybrids to “*T*-bias” or “*K-*bias” in the allotetraploids than from “*T-*bias” or “*K*-bias” to “Non-bias.”

**Table 2:** The number of genes showing homoeolog expression bias in the F_1_ hybrids and the allotetraploids, *Phegopteris decursivepinnata*.

	F_1K_ hybrid	F_1T_ hybrid	Allotetraploid
**Non-bias**	6801	7529	6363
***K*-bias**	1339	991	1558
***T*-bias**	1400	1020	1619

**Figure 5:**
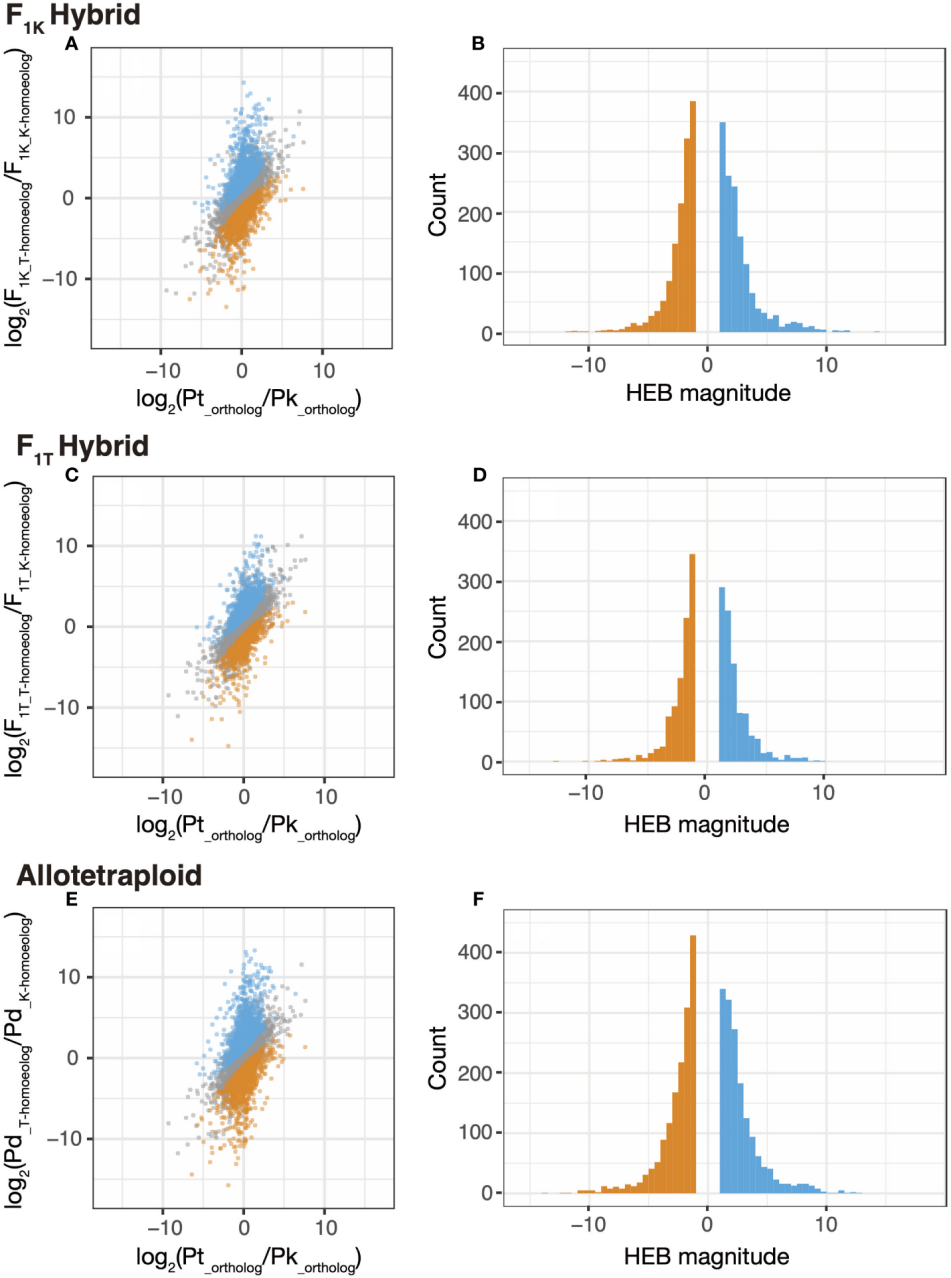
Homoeolog expression bias (HEB) in the F_1K_ hybrids, the F_1T_ hybrids, and the allotetraploids, *Phegopteris decursivepinnata*. **(A, B)** the F_1K_ hybrids, **(C, D)** F_1T_ hybrids. **(E, F)** Allotetraploids. **(A, C, E)** Scatter plots of gene pairs showing HEB in F_1K_ hybrids **(A)**, F_1T_ hybrids **(C)**, and allotetraploids **(E)**. The horizontal axis is log_2_(fold change (FC)) between the orthologous genes of *P. koreana* and *P. taiwaniana* and the vertical axis is log_2_(FC) of the homoeologous genes in F_1_ hybrids or allotetraploids; gray, blue, and orange points indicate gene pairs exhibiting no HEB, *T-*bias, and *K-*bias, respectively. **(B, D, F)** Histograms showing the “magnitude” of HEB calculated from the formula; log_2_((F_1K_T-homoeolog/_F_1K_K-homoeolog_)/(Pt__ortholog/_Pk__ortholog_)) **(B)**, log_2_((F_1T_T-homoeolog/_F_1T_K-homoeolog_)/(Pt__ortholog/_Pk__ortholog_)) **(D)**, and log_2_((Pd__T-homoeolog/_Pd__K-homoeolog_)/(Pt__ortholog/_Pk__ortholog_)) **(F)**. Blue and orange bars indicate *T-* and *K-*biased gene pairs, respectively. Pd, *P. decursivepinnata*; Pk, *P. koreana*; Pt, *P. taiwaniana*.

To examine the differences in strength of the HEB between the F_1K_ hybrids and the allotetraploids, the relative expression levels of the homoeolog pairs in the F_1_ hybrids and the allotetraploids were compared with the orthologous gene pairs between the parental species (**Figure 5**). The F_1_ hybrids and the allotetraploids showed a similar trend whereas both the *T-*biased and *K-*biased gene pairs showed a similar distribution in the scatter plot and histogram (**Figure 5**). In the allotetraploids, along with increases in both subgenome-biased gene pairs, the strength of both biases also increased (**Table 3**; **Figure 5**). The number of genes with a “magnitude” > |10| for *T-* and *K-*biased gene pairs increased from nine in the F_1K_ hybrids and none in the F_1T_ hybrids to 11 in the allotetraploids for *T*-biased genes, and five in the F_1K_ hybrids and two in the F_1T_ hybrids to 13 in the allotetraploids for *K*-biased genes, respectively (**Table 3**). Additionally, the mean values of “magnitude” for *T*- and *K*-biased genes were also amplified from 2.72 in the F_1K_ hybrids and 2.39 in the F_1T_ hybrids to 2.83 in the allotetraploids for *T*-biased genes, and −2.36 in the F_1K_ hybrids and −2.19 in the F_1T_ hybrids to −2.64 in the allotetraploids for *K*-biased genes (**Table 3**). Finally, we explored the functional differences associated with HEB, showing both *T*-biased and *K*-biased expression in the F_1K_ hybrids and the allotetraploids. Among the five *K*-biased genes and nine *T*-biased genes in the the F_1K_ hybrids, and 13 *K*-biased genes and 11 *T*-biased genes in the allotetraploids (a “magnitude” >|10|), four, nine, 11 and nine genes were successfully identified using blast analysis against the *Arabidopsis* protein sequences for *K*- and *T*-bias of the F_1K_ hybrids and the allotetraploids, respectively (**Supplementary Tables S4–S7**).

**Table 3:** Magnitude of homoeolog expression bias in the F_1_ hybrids and the allotetraploids, *Phegopteris decursivepinnata*.

	# of T-biased genes with a “magnitude” >10	# of K-biased genes with a “magnitude” <−10	mean & median of “magnitude” in T-biased genes	mean & median of “magnitude” in K-biased genes
F_1K_ hybrid	9	5	2.72, 2.20	−2.36, -1.94
F_1T_ hybrid	0	2	2.39, 1.94	−2.19, -1.79
Allotetraploid	11	13	2.83, 2.27	−2.64, -2.1

A “magnitude” value was calculated by the formula: log_2_((F_1_T-homoeolog_/F_1_K-homoeolog_)/(Pt__ortholog_/Pk__ortholog_)) for F_1_ hybrids, and log_2_((Pd__T-homoeolog_/Pd__K-homoeolog_)/(Pt__ortholog_/Pk__ortholog_)) for allotetraploids, *Phegopteris decursivepinnata*. Pd, *P. decursivepinnata*; Pk, *P. koreana*; Pt, *P. taiwaniana*.

## 4 Discussion

### 4.1 Changes in gene expression levels between the F_1_ hybrids and the allotetraploid *Phegopteris decursivepinnata*

Remarkable changes were observed in the gene expression levels of the F_1_ hybrids and the allotetraploids when compared to the parental species (**Figure 1**). Notably, more than half of genes (71.14%) showing non-additive expression in the F_1K_ hybrids were also detected as non-additive in allotetraploids (**Figure 2**). These findings suggest that the merger of divergent genomes through hybridization itself had a substantial impact on gene expression changes, which is consistent with previous studies (Hegarty et al., 2006; Chaudhary et al., 2009; Flagel and Wendel, 2010; Buggs et al., 2011; Duan et al., 2023). However, we also observed substantial differences in expression levels between the F_1_ hybrids and the allotetraploids. In the allotetraploids, the proportion of genes categorized as “No Change” was decreased compared to those in the F_1_ hybrids (77.4% in the allotetraploid vs. 80.1% and 81.8% in the F_1_ hybrids).

The gene showing transgressive expression was more frequent in the allotetraploids than in the F_1_ hybrids (1,188 genes in the allopolyploids vs. 926 and 785 genes in the F_1_ hybrids) (**Figure 1**). Interestingly, although very low rates of transgressive expression patterns in allopolyploid fern species (approximately 1% or less in both “Transgressive Up-regulation” and “Transgressive Down-regulation”) have been reported in *Polypodium hesperium* (Sigel et al., 2019), the proportion in this study is rather consistent with the reports from allopolyploids of flowering plants: 12.5% in this study, 8.9–10.9% in *Achillea* (Chen et al. 2021), 16–18% in *Coffea* (Bardil et al., 2011), 13.2–30.4% in *Gossypium* (Flagel and Wendel, 2010) and 5.5–8.9% in *Tragopogon* (Shan et al., 2020). Tracing the category transition of genes between the F_1K_ hybrids and the allotetraploids, the transition from the category “No-Change” to “Transgressive Down-regulation” mostly accounted for the increase in the number of genes showing transgressive expression (**Figure 2**). This result indicates that additional expression changes that had occurred after polyploidization contributed to the higher rate of transgressive expression genes in the allotetraploids. A similar phenomenon including the category transition from “No change” to transgressive expression has been reported in angiosperms, and was likely to be caused by epigenetic modification or *cis-* and *trans*-regulatory evolution (Flagel and Wendel, 2010; Hu and Wendel, 2019).

ELD is a well-known phenomenon observed in numerous allopolyploids as demonstrated in several previous studies (Rapp et al., 2009; Flagel and Wendel, 2010; Bardil et al., 2011; Sigel et al., 2019; Chen et al., 2021). In this study, a substantial number of genes showing “*K*-dominance” or “*T*-dominance” in the F_1K_ hybrids was also highly conserved in the allotetraploids: 67.3% in “*K*-dominance” and 63.3% in “*T*-dominance” (**Figure 2**). Therefore, hybridization plays a major role in shaping the pattern of ELD in this study. In many allopolyploid species, unbalanced ELD has been reported, with a tendency for expression levels of more genes to resemble those of one parental species (Rapp et al., 2009; Wu et al., 2018; Chen et al., 2021). Similarly, in the allopolyploid fern *Polypodium*, almost all genes showing ELD are reportedly biased toward one parental species (Sigel et al., 2019). In this study, however, although the number of genes showing “*T*-dominance” was slightly higher than those showing “*K-*dominance” in both the F_1K_ hybrids (5.6% vs. 4.3%) and the F_1T_ hybrids (6.0% vs. 3.8%), the allotetraploids showed the same extent in the proportion of genes exhibiting either “*T*-dominance” and “*K*-dominance” (5.1% vs. 4.8%). This pattern of ELD in the allotetraploids was shaped by larger outflows from “*T*-dominance” to other categories than inflows and larger inflows from other categories into “*K*-dominance” than outflows (**Figure 2**). This suggests that the hybridization between *Phegopteris koreana* and *P. taiwaniana* contributed to “*T*-dominance”, and the alleviation of “*T*-dominance” and the activation of “*K*-dominance” occurred over evolutionary time after polyploidization.

### 4.2 Homoeolog expression bias in the F_1_ hybrids and the allotetraploid *Phegopteris decursivepinnata*

HEB is the phenomenon where F_1_ or allopolyploid homoeologous gene pairs exhibit expression ratios that differ from those of orthologous gene pairs of their parent species (Yoo et al., 2013). HEB has been frequently documented in allopolyploid species (Chaudhary et al., 2009; Koh et al., 2010; Edger et al., 2017; Boatwright et al., 2018; Sigel et al., 2019; Boatwright et al., 2021; Chen et al., 2021; Vasudevan et al., 2023). In this study, 21.1–33.3% of the 9,540 gene pairs showed HEB in both the F_1_ hybrids and the allotetraploids (**Table 2**). Importantly, a significant number of genes displaying HEB were shared between the F_1_ hybrids and the allotetraploids. More than half of the genes showing HEB in the F_1_ hybrids (60.5%) were also identified with HEB in the allotetraploids. The transition from “*K-*bias” to “*T-*bias” or vice versa between the F_1_ hybrids and the allotetraploids was infrequently observed (**Figure 4**). These findings demonstrate that HEB primarily originated in the response to hybridization before the occurrence of polyploidization. However, there was also a substantial change in the pattern between the F_1_ hybrids and the allotetraploids—the intensification of HEB in the allotetraploids compared to the F_1_ hybrids. This intensification was evident not only in the number of genes showing HEB but also in the “magnitude” of bias for both “*K*-bias” and “*T*-bias” in the allotetraploids (see **Tables 2**, **3**; **Figure 5**). These changes resulted in the lowest kurtosis in the distribution of the expression level ratio between homoeologs in the allotetraploids (**Table 1**; **Figure 3**). Therefore, this observation suggests that HEB gradually intensified in the number of genes and the “magnitude” of bias after polyploidization. Unbalanced HEB is commonly reported across allopolyploids (Chaudhary et al., 2009; Koh et al., 2010; Edger et al., 2017; Sigel et al., 2019; Chen et al., 2021; Vasudevan et al., 2023). In this study, there was no apparent bias toward either parent in the F_1_ hybrids, and the allotetraploids (1,339 “*K*-bias” vs. 1,400 “*T*-bias” in the F_1K_ hybrids, 1,020 vs. 991 in the F_1T_ hybrids, and 1,619 vs. 1,558 in the allotetraploids) (**Table 2**). This pattern of HEB aligns with the previous reports of allopolyploids exhibiting a balanced HEB (Boatwright et al., 2018; Boatwright et al., 2021; Burns et al., 2021; Wu et al., 2021). The balanced HEB obtained in this study contrasts with the previous study focusing on allotetraploid fern, which reported an exceptionally strong HEB towards one parent (744–923 genes vs. 39–100 genes) (Sigel et al., 2019). Therefore, the strong unbalanced HEB observed in the previous study is not a common pattern in transcriptomic change after allopolyploidization among ferns.

The HEB is expected to be partly attributed to cytonuclear gene coordination (Sharbrough et al., 2017), although its significance for gene expression patterns in allopolyploids remains inconsistent among studies (Gong et al., 2012; Gong et al., 2014; Sehrish et al., 2015; Ferreira De Carvalho et al., 2019). In allopolyploid plants, the nuclear genome is contributed from both parental species, but organellar genomes are inherited only from maternal parents, including ferns (Gastony and Yatskievych, 1992; reviewed in Kuo L.-Y. et al., 2018). Therefore, cytonuclear coordination should occur following hybridization, where the expression of nuclear-encoded organelle-targeted genes is biased toward the maternal parent, or the function of the paternal homoeolog is lost (Sharbrough et al., 2017). Given that the maternal parent of *Phegopteris decursivepinnta* is *P. koreana* (Fujiwara et al., 2021), it is suggested that cytonuclear interactions might favor higher expressions of organelle-targeted homoeologs derived from *P. koreana.* In this study, among genes exhibiting extremely strong HEB (“magnitude” > |10|), whereas one out of five *K*-biased genes and two out of nine *T*-biased genes in the F_1K_ hybrids were associated with chloroplast and mitochondria-related functions (**Supplementary Tables S6, S7**), four out of 13 *K*-biases genes and four out of 11 *T*-biased genes in the allotetraploids were detected to be functionally related to organelle (**Supplementary Tables S4, S5**). Two of four organelle-related *K*-biased genes detected in the allotetraploids are orthologue to *AtRBCX1* (AT4G04330), which functions in RuBisCO assembly (Kolesiński et al., 2011), and to *NDU9/B14.5b*, a subunit of mitochondrial Complex I (Subrahmanian et al., 2016) (**Supplementary T** **able S4**). Consistent with our initial prediction, our results support the idea that cytonuclear coordination favors the higher expression of the maternal homologue in organelle-targeted genes, particularly those directly related to organelle function and structure. Therefore, this finding indicates that the increased HEB in the allotetraploids observed in this study can be partly explained by the concerted action between the duplicated nuclear genome and the increased copy of organelle genomes in cells.

### 4.3 The absence of subgenome dominance in *Phegopteris decursivepinnata* and its implication for genome evolution in ferns

Our results showed that neither ELD nor HEB showed a pronounced bias toward one of the subgenomes in the F_1_ hybrids and the natural allotetraploids, *Phegopteris decursivepinnata*. The pattern observed in *P. decursivepinnata* can be interpreted as the absence of subgenome dominance when compared with the previous studies (Flagel and Wendel, 2010; Edger et al., 2017; Bird et al., 2018; Sigel et al., 2019; Chen et al., 2021; Glombik et al., 2021; Vasudevan et al., 2023). The absence of subgenome dominance in *P. decursivepinnata* contrasts with the extreme case of subgenome dominance observed in *Polypodium hesperium*, the only fern species for which DEG analysis has been conducted to date (Sigel et al., 2019). The difference in transcriptomic consequence of allopolyploidy between the two ferns may be explained by two possible reasons.

Firstly, the age of allopolyploid ferns may reflect the degree of subgenome dominance. In other words, it is likely that subgenome dominance has not extensively proceeded in *Phgopteris decursivepinnata* due to its relatively recent origin. The previous phylogenetic analysis showed no genetic differentiation from parental species for cpDNA gene (*rbcL*) and nuclear DNA (*pgiC*), indicating the recent origin of the allotetraploid species (Fujiwara et al., 2021). However, because the parental species currently do not co-occur due to their ecological differentiation, the allopolyploid speciation is not an ongoing process, but rather more likely to have occurred during the climatic oscillations in the Quaternary, as suggested for the other allopolyploid ferns in Japan (Fujiwara and Watano, 2020; Fujiwara et al., 2022). However, the allopolyploid fern showing strong subgenome dominance, *Polypodium hesperium* is also considered to have originated very recently during glacial cycles in the Pleistocene (Sigel et al., 2014). Therefore, the difference in the degree of subgenome dominance between the two allopolyploid ferns with recent origins cannot be attributed to the difference in their age since polyploidization. Secondly, the degree of subgenome dominance may be linked to genomic differentiation between parental species, known as parental legacy (Buggs et al., 2014). In other words, the genomic properties inherited from the parental species may predetermine the tendency of subgenome dominance. Indeed, in allopolyploid species that exhibit strong subgenome dominance, the bias toward one subgenome is already evident in the F_1_ hybrids and the first generation of synthetic allopolyploids and subsequently tends to be strengthened in the later generations of allopolyploids (Flagel and Wendel, 2010; Edger et al., 2017; Bird et al., 2021; Vasudevan et al., 2023). Increasing evidence suggests that one significant aspect of the genomic difference between parental species that contributes to subgenome dominance is the variation in the abundance of transposable elements (TEs) between them (Bird et al., 2018; Alger and Edger, 2020). TEs can be activated during allopolyploidization, but subsequently, most TEs are rapidly silenced. This process affects the expression of the neighboring coding genes, resulting in a lower expression or silencing in the homoeologous genes of a subgenome possessing more TEs when compared with another subgenome. According to the hypothesis, the observed difference in subgenome dominance between the two allopolyploid ferns may be explained by the difference between the two species in genomic differentiation in TE abundance between the parental species. Future studies will require an investigation into the differences in the genomic composition of these species by focusing on the abundance of TEs.

Although we found the contrasting result of subgenome dominance between two allopolyploid ferns, several lines of genomic evidence can predict that the absence of subgenome dominance may be more common, particularly in homosporous ferns. Unlike angiosperms, ferns are hypothesized to exhibit low TE activity, and this is supported by the old insertion time of LTR retrotransposons (Baniaga and Barker, 2019) and genome size stability over long periods (Schneider et al., 2015; Clark et al., 2016; Fujiwara et al., 2023). Therefore, ferns may maintain a highly similar genomic landscape (Szövényi et al., 2021; Huang et al., 2022), even between species from different genera (Rothfels et al., 2015), and the relatively less divergent genome in ferns could potentially generate subtle or absence of subgenome dominance in most homosporous fern allopolyploids, as observed in this study. Furthermore, subgenome dominance is hypothesized to trigger a biased fractionation of the recessive subgenome, leading to rapid diploidization and genome-downsizing in polyploid genomes (Cheng et al., 2018; Wendel et al., 2018; Alger and Edger, 2020). In ferns, however, it is traditionally believed that the diploidization process is slower when compared with angiosperms (Barker, 2013). Instead of the physical loss of genes, the diploidization in ferns is hypothesized to involve gene silencing, leading to slow rates of chromosome reduction and genome downsizing (Haufler, 2014). The limited empirical evidence based on genome sequencing also supports this hypothesis on slow genome evolution in homosporous ferns (Huang et al., 2022; Zhong et al., 2022), with one exception (Marchant et al., 2022). Therefore, the observed absence of subgenome dominance in *Phegopteris decursivepinnata* is consistent with the slow genome downsizing in ferns and could partially explain the process of post-polyploid evolution seen in the broad range of homosporous ferns. Also in homosporous lycophyte, a lineage of land plants considered to exhibit a similar genome evolution as homosporous ferns (Sessa and Der, 2016), a recent finding from ancient allotetraploid *Huperzia* showed a limited subgenome dominance and slow genome evolution following polyploidization (Li et al., 2023), which support our prediction. To test the validity of our prediction, we still need to examine transcriptomic change and the pattern of genomic differentiation including TE abundance in more diverse allopolyploid fern species.

### 4.4 Limitations in this study and future directions

In this study, we successfully demonstrated the dynamics of gene expression change between the F_1_ hybrids and the allotetraploids of *Phegopteris* fern, highlighting the absence of subgenome dominance in the allotetraploid. Nevertheless, it is essential to note several limitations in our study design and technical approaches that need to be addressed in future investigations.

Firstly, one of the limitations is the relatively limited number of genes examined in this study. We targeted 9,540 genes co-expressed among the parental species, the F_1_ hybrids, and the allotetraploids. This represents less than half of the total number of protein-coding genes reported in homosporous ferns (36,857 genes in *Ceratopteris*, Marchant et al., 2022; 31,244 genes in *Adiantum*, Fang et al., 2022). However, this is because the necessity of focusing on co-expressed genes arose from the absence of available genome sequence data for *Phegopteris* fern and the specific goal of tracing the transition of genes exhibiting ELD and HEB from the F_1_ hybrids to the allotetraploids. Although our study successfully provided a robust overview of the transcriptomic changes in the allotetraploids as evidenced by the consistency across three datasets with TPM filtering thresholds of > 0, > 0.5, and > 1.0 (**Supplementary Figures S4–S7**), it should be noted that our results are solely based on co-expressed genes and thus might have led to the oversight of critical changes, such as gene silencing, and reactivation of genes suppressed in the parental species. To comprehensively examine genome-wide expression changes following allopolyploidization, future studies should consider analyzing transcriptome data using parental genome sequences as references.

Secondly, this study is solely based on gene expression in leaf tissues, possibly overlooking tissue-specific expression differences. Previous studies comparing homoeolog gene expression among different tissues reported substantial differentiation of homoeolog expression bias in direction and expression level for each gene among the examined tissues (Adams et al., 2003; Buggs et al., 2010; Huang et al., 2022). On the other hand, recent studies focusing on the whole transcriptome-wide pattern of homoeolog gene expression suggest that the overall trend of homoeolog expression bias is highly conserved among different tissues (Edger et al., 2017; Griffiths et al., 2019; Chen et al., 2021; Vasudevan et al., 2023). Therefore, while the trend obtained in this study could be consistent among other tissues, we might have missed key changes in homoeolog gene expression occurring in different tissues, such as tissue-specific silencing and tissue-specific homoeolog bias, as reported in other studies. Thus, in future studies, it is required to examine gene expression patterns using different tissue types of *Phegopteris* species.

Lastly, the pattern of gene expression in the allotetraploids might change in response to environmental conditions (Shimizu, 2022). In *Cardamine*, studies have shown that allopolyploids in each dry and humid environment tend to exhibit gene expression patterns resembling those of each parental species specialized for each environment, which enables the allopolyploids to obtain adaptive plasticity to diverse niches (Shimizu-Inatsugi et al., 2016; Akiyama et al., 2021). *Phegopteris decursivepinnata* has a wider distribution than those of its parental species (Fujiwara et al., 2021) and thus is expected to have a higher tolerance to a wide range of environmental conditions than its parental species. Given that, it is likely that *P. decursivepinnata* could also show different patterns of homoeolog gene expression from that observed in this study according to different environmental conditions. Thus, it is essential to trace the change in the pattern of gene expression in response to different environmental conditions in this species.

## Data availability statement

The raw sequence data used in this study were deposited in the DDBJ sequence Read Archive (DRA) under the accession numbers (DRR498230–DRR498244).

## Author contributions

NK: Conceptualization, Data curation, Formal analysis, Methodology, Writing – original draft, Writing – review & editing. TY: Data curation, Formal analysis, Writing – review & editing. SA: Data curation, Formal analysis, Writing – review & editing. YW: Conceptualization, Project administration, Supervision, Writing – review & editing. TF: Conceptualization, Formal Analysis, Funding acquisition, Project administration, Supervision, Writing – original draft, Writing – review & editing.

## References

[B1] Adams K. L. Cronn R. Percifield R. Wendel J. F. (2003). Genes duplicated by polyploidy show unequal contributions to the transcriptome and organ-specific reciprocal silencing. Proc. Natl. Acad. Sci. U.S.A. 100, 4649–4654. doi: 10.1073/pnas.0630618100 12665616 PMC153610

[B3] Akiyama R. Sun J. Hatakeyama M. Lischer H. E. Briskine R. V. Hay A.. (2021). Fine-scale empirical data on niche divergence and homeolog expression patterns in an allopolyploid and its diploid progenitor species. New Phytol. 229, 3587–3601. doi: 10.1111/nph.17101 33222195 PMC7986779

[B2] Alger E. I. Edger P. P. (2020). One subgenome to rule them all: underlying mechanisms of subgenome dominance. Curr. Opin. Plant Biol. 54, 108–113. doi: 10.1016/j.pbi.2020.03.004 32344327

[B4] Baniaga A. E. Barker M. S. (2019). Nuclear genome size is positively correlated with median LTR-RT insertion time in fern and lycophyte genomes. Am. Fern J. 109, 248. doi: 10.1640/0002-8444-109.3.248

[B5] Bardil A. De Almeida J. D. Combes M. C. Lashermes P. Bertrand B. (2011). Genomic expression dominance in the natural allopolyploid *Coffea arabica* is massively affected by growth temperature. New Phytol. 192, 760–774. doi: 10.1111/j.1469-8137.2011.03833.x 21797880

[B6] Barker M. S. (2013). “Karyotype and genome evolution in pteridophytes,” in Plant genome diversity, vol. 2 . Eds. Leitch I. J. Greilhuber J. Dolezel J. Wendel J. F. (Berlin: Springer), 245–253. doi: 10.1007/978-3-7091-1160-4_15

[B7] Barker M. S. Arrigo N. Baniaga A. E. Li Z. Levin D. A. (2016). On the relative abundance of autopolyploids and allopolyploids. New Phytol 210, 391–398. doi: 10.1111/nph.13698 26439879

[B8] Beaulieu J. M. Leitch I. J. Patel S. Pendharkar A. Knight C. A. (2008). Genome size is a strong predictor of cell size and stomatal density in angiosperms. New Phytol. 179, 975–986. doi: 10.1111/j.1469-8137.2008.02528.x 18564303

[B9] Birchler J. A. Yang H. (2022). The multiple fates of gene duplications: Deletion, hypofunctionalization, subfunctionalization, neofunctionalization, dosage balance constraints, and neutral variation. Plant Cell 34, 2466–2474. doi: 10.1093/plcell/koac076 35253876 PMC9252495

[B10] Bird K. A. Niederhuth C. E. Ou S. Gehan M. Pires J. C. Xiong Z.. (2021). Replaying the evolutionary tape to investigate subgenome dominance in allopolyploid *Brassica napus* . New Phytol. 230, 354–371. doi: 10.1111/nph.17137 33280122 PMC7986222

[B11] Bird K. A. VanBuren R. Puzey J. R. Edger P. P. (2018). The causes and consequences of subgenome dominance in hybrids and recent polyploids. New Phytol. 220, 87–93. doi: 10.1111/nph.15256 29882360

[B12] Boatwright J. L. McIntyre L. M. Morse A. M. Chen S. Yoo M.-J. Koh J.. (2018). A robust methodology for assessing differential homeolog contributions to the transcriptomes of allopolyploids. Genetics 210, 883–894. doi: 10.1534/genetics.118.301564 30213855 PMC6218233

[B13] Boatwright J. L. Yeh C.-T. Hu H.-C. Susanna A. Soltis D. E. Soltis P. S.. (2021). Trajectories of homoeolog-specific expression in allotetraploid *Tragopogon castellanus* populations of independent origins. Front. Plant Sci. 12. doi: 10.3389/fpls.2021.679047 PMC8261302 34249049

[B14] Bolger A. M. Lohse M. Usadel B. (2014). Trimmomatic: a flexible trimmer for Illumina sequence data. Bioinformatics 30, 2114–2120. doi: 10.1093/bioinformatics/btu170 24695404 PMC4103590

[B15] Buggs R. J. Elliott N. M. Zhang L. Koh J. Viccini L. F. Soltis D. E.. (2010). Tissue-specific silencing of homoeologs in natural populations of the recent allopolyploid *Tragopogon mirus* . New Phytol. 186, 175–183. doi: 10.1111/j.1469-8137.2010.03205.x 20409177

[B16] Buggs R. J. A. Wendel J. F. Doyle J. J. Soltis D. E. Soltis P. S. Coate J. E. (2014). The legacy of diploid progenitors in allopolyploid gene expression patterns. Phil. Trans. R. Soc B 369, 20130354. doi: 10.1098/rstb.2013.0354 24958927 PMC4071527

[B17] Buggs R. J. A. Zhang L. Miles N. Tate J. A. Gao L. Wei W.. (2011). Transcriptomic shock generates evolutionary novelty in a newly formed, natural allopolyploid plant. Curr.Biol 21, 551–556. doi: 10.1016/j.cub.2011.02.016 21419627

[B18] Burns R. Mandáková T. Gunis J. Soto-Jiménez L. M. Liu C. Lysak M. A.. (2021). Gradual evolution of allopolyploidy in *Arabidopsis suecica* . Nat. Ecol. Evol. 5, 1367–1381. doi: 10.1038/s41559-021-01525-w 34413506 PMC8484011

[B19] Chaudhary B. Flagel L. Stupar R. M. Udall J. A. Verma N. Springer N. M.. (2009). Reciprocal silencing, transcriptional bias and functional divergence of homeologs in polyploid cotton (*Gossypium*). Genetics 182, 503–517. doi: 10.1534/genetics.109.102608 19363125 PMC2691759

[B20] Chen D. Yan P.-C. Guo Y.-P. (2021). Imprints of independent allopolyploid formations on patterns of gene expression in two sibling yarrow species (*Achillea*, Asteraceae). BMC Genomics 22, 264. doi: 10.1186/s12864-021-07566-6 33849436 PMC8045213

[B21] Chen H. Fang Y. Zwaenepoel A. Huang S. Van De Peer Y. Li Z. (2023). Revisiting ancient polyploidy in leptosporangiate ferns. New Phytol. 237, 1405–1417. doi: 10.1111/nph.18607 36349406 PMC7614084

[B22] Cheng F. Wu J. Cai X. Liang J. Freeling M. Wang X. (2018). Gene retention, fractionation and subgenome differences in polyploid plants. Nat. Plants 4, 258–268. doi: 10.1038/s41477-018-0136-7 29725103

[B23] Clark J. Hidalgo O. Pellicer J. Liu H. Marquardt J. Robert Y.. (2016). Genome evolution of ferns: evidence for relative stasis of genome size across the fern phylogeny. New Phytol. 210, 1072–1082. doi: 10.1111/nph.13833 26756823

[B24] De Smet R. Adams K. L. Vandepoele K. Van Montagu M. C. E. Maere S. Van De Peer Y. (2013). Convergent gene loss following gene and genome duplications creates single-copy families in flowering plants. Proc. Natl. Acad. Sci. U.S.A. 110, 2898–2903. doi: 10.1073/pnas.1300127110 23382190 PMC3581894

[B25] Duan T. Sicard A. Glémin S. Lascoux M. (2023). Expression pattern of resynthesized allotetraploid *Capsella* is determined by hybridization, not whole-genome duplication. New Phytol. 237, 339–353. doi: 10.1111/nph.18542 36254103 PMC10099941

[B26] Edger P. P. Smith R. McKain M. R. Cooley A. M. Vallejo-Marin M. Yuan Y.. (2017). Subgenome dominance in an interspecific hybrid, synthetic allopolyploid, and a 140-year-old naturally established neo-allopolyploid monkeyflower. Plant Cell 29, 2150–2167. doi: 10.1105/tpc.17.00010 28814644 PMC5635986

[B27] Emms D. M. Kelly S. (2019). OrthoFinder: phylogenetic orthology inference for comparative genomics. Genome Biol. 20, 1–14. doi: 10.1186/s13059-019-1832-y 31727128 PMC6857279

[B28] Fang Y. Qin X. Liao Q. Du R. Luo X. Zhou Q.. (2022). The genome of homosporous maidenhair fern sheds light on the euphyllophyte evolution and defenses. Nat. Plants 8, 1024–1037. doi: 10.1038/s41477-022-01222-x 36050462 PMC7613604

[B29] Ferreira De Carvalho J. Lucas J. Deniot G. Falentin C. Filangi O. Gilet M.. (2019). Cytonuclear interactions remain stable during allopolyploid evolution despite repeated whole-genome duplications in *Brassica* . Plant J. 98, 434–447. doi: 10.1111/tpj.14228 30604905

[B30] Flagel L. E. Wendel J. F. (2010). Evolutionary rate variation, genomic dominance and duplicate gene expression evolution during allotetraploid cotton speciation. New Phytol. 186, 184–193. doi: 10.1111/j.1469-8137.2009.03107.x 20002320

[B31] Fujiwara T. Egashira T. Gutiérrez-Ortega J. S. Hori K. Ebihara A. Watano Y. (2022). Establishment of an allotetraploid fern species, *Lepisorus yamaokae* Seriz., between two highly niche-differentiated parental species. Am. J. Bot. 109, 1456–1471. doi: 10.1002/ajb2.16043 35938973

[B32] Fujiwara T. Liu H. Meza-Torres E. I. Morero R. E. Vega A. J. Liang Z.. (2023). Evolution of genome space occupation in ferns: linking genome diversity and species richness. Ann. Bot. 131, 59–70. doi: 10.1093/aob/mcab094 34259813 PMC9904345

[B33] Fujiwara T. Ogiso J. Ishii S. Togo K. Nakato N. Serizawa S.. (2021). Species delimitation in the *Phegopteris decursivepinnata* polyploid species complex (Thelypteridaceae). Acta Phytotax. Geobot. 72, 205–226. doi: 10.18942/apg.202102

[B34] Fujiwara T. Watano Y. (2020). Independent allopatric polyploidizations shaped the geographical structure and initial stage of reproductive isolation in an allotetraploid fern, *Lepisorus nigripes* (Polypodiaceae). PloS One 15, e0233095. doi: 10.1371/journal.pone.0233095 32433707 PMC7239481

[B35] Gao B. Chen M. Li X. Liang Y. Zhang D. Wood A. J.. (2022). Ancestral gene duplications in mosses characterized by integrated phylogenomic analyses. J. Syst. Evol. 60, 144–159. doi: 10.1111/jse.12683

[B36] Gastony G. J. Yatskievych G. (1992). Maternal inheritance of the chloroplast and mitochondrial genomes in cheilanthoid ferns. Am. J. Bot. 79, 716–722. doi: 10.1002/j.1537-2197.1992.tb14613.x

[B37] Glombik M. Copetti D. Bartos J. Stoces S. Zwierzykowski Z. Ruttink T.. (2021). Reciprocal allopolyploid grasses (*Festuca* × *Lolium*) display stable patterns of genome dominance. Plant J. 107, 1166–1182. doi: 10.1111/tpj.15375 34152039 PMC8518873

[B38] Gong L. Olson M. Wendel J. F. (2014). Cytonuclear evolution of Rubisco in four allopolyploid lineages. Mol. Biol. Evol. 31, 2624–2636. doi: 10.1093/molbev/msu207 25015644 PMC4166922

[B39] Gong L. Salmon A. Yoo M.-J. Grupp K. K. Wang Z. Paterson A. H.. (2012). The cytonuclear dimension of allopolyploid evolution: an example from cotton using Rubisco. Mol. Biol. Evol. 29, 3023–3036. doi: 10.1093/molbev/mss110 22490824

[B40] Grabherr M. G. Haas B. J. Yassour M. Levin J. Z. Thompson D. A. Amit I.. (2011). Full-length transcriptome assembly from RNA-Seq data without a reference genome. Nat. Biotechnol. 29, 644–652. doi: 10.1038/nbt.1883 21572440 PMC3571712

[B41] Griffiths A. G. Moraga R. Tausen M. Gupta V. Bilton T. P. Campbell M. A.. (2019). Breaking free: the genomics of allopolyploidy-facilitated niche expansion in white clover. Plant Cell 31, 1466–1487. doi: 10.1105/tpc.18.00606 31023841 PMC6635854

[B42] Grover C. E. Gallagher J. P. Szadkowski E. P. Yoo M. J. Flagel L. E. Wendel J. F. (2012). Homoeolog expression bias and expression level dominance in allopolyploids. New Phytol. 196, 966–971. doi: 10.1111/j.1469-8137.2012.04365.x 23033870

[B43] Haufler C. H. (2014). Ever since Klekowski: Testing a set of radical hypotheses revives the genetics of ferns and lycophytes. Am. J. Bot. 101, 2036–2042. doi: 10.3732/ajb.1400317 25480700

[B44] Haufler C. H. Pryer K. M. Schuettpelz E. Sessa E. B. Farrar D. R. Moran R.. (2016). Sex and the single gametophyte: Revising the homosporous vascular plant life cycle in light of contemporary research. BioScience 66, 928–937. doi: 10.1093/biosci/biw108

[B45] Hegarty M. J. Barker G. L. Wilson I. D. Abbott R. J. Edwards K. J. Hiscock S. J. (2006). Transcriptome shock after interspecific hybridization in *Senecio* is ameliorated by genome duplication. Curr. Biol. 16, 1652–1659. doi: 10.1016/j.cub.2006.06.071 16920628

[B46] Hegarty M. J. Hiscock S. J. (2008). Genomic clues to the evolutionary success of polyploid plants. Curr. Biol. 18, R435–R444. doi: 10.1016/j.cub.2008.03.043 18492478

[B47] Hu G. Wendel J. F. (2019). Cis–trans controls and regulatory novelty accompanying allopolyploidization. New Phytol. 221, 1691–1700. doi: 10.1111/nph.15515 30290011

[B48] Huang C. Qi X. Chen D. Qi J. Ma H. (2020). Recurrent genome duplication events likely contributed to both the ancient and recent rise of ferns. J. Integr. Plant Biol. 62, 433–455. doi: 10.1111/jipb.12877 31628713

[B49] Huang X. Wang W. Gong T. Wickell D. Kuo L.-Y. Zhang X.. (2022). The flying spider-monkey tree fern genome provides insights into fern evolution and arborescence. Nat. Plants 8, 500–512. doi: 10.1038/s41477-022-01146-6 35534720 PMC9122828

[B50] Kaltenegger E. Ober D. (2015). Paralogue interference affects the dynamics after gene duplication. Trends Plant Sci. 20, 814–821. doi: 10.1016/j.tplants.2015.10.003 26638775

[B51] Kawakami S. M. Kato J. Kawakami S. (2019). Meiosis of dihaploid *Thelypteris decursive-pinnata* produced artificially by induced apogamy. Cytologia 84, 319–322. doi: 10.1508/cytologia.84.319

[B52] Koh J. Soltis P. S. Soltis D. E. (2010). Homeolog loss and expression changes in natural populations of the recently and repeatedly formed allotetraploid *Tragopogon mirus* (Asteraceae). BMC Genomics 11, 97. doi: 10.1186/1471-2164-11-97 20141639 PMC2829515

[B53] Kolesiński P. Piechota J. Szczepaniak A. (2011). Initial characteristics of RbcX proteins from *Arabidopsis thaliana* . Plant Mol. Biol. 77, 447–459. doi: 10.1007/s11103-011-9823-8 21922322 PMC3195260

[B54] Kuo L.-Y. Tang T.-Y. Li F.-W. Su H.-J. Chiou W.-L. Huang Y.-M.. (2018). Organelle genome inheritance in *Deparia* ferns (Athyriaceae, Aspleniineae, Polypodiales). Front. Plant Sci. 9. doi: 10.3389/fpls.2018.00486 PMC5932399 29755486

[B55] Kuo T. Frith M. C. Sese J. Horton P. (2018). EAGLE: explicit alternative genome likelihood evaluator. BMC Med. Genomics 11, 28. doi: 10.1186/s12920-018-0342-1 29697369 PMC5918433

[B56] Landis J. B. Soltis D. E. Li Z. Marx H. E. Barker M. S. Tank D. C.. (2018). Impact of whole-genome duplication events on diversification rates in angiosperms. Am. J. Bot. 105, 348–363. doi: 10.1002/ajb2.1060 29719043

[B57] Langmead B. Salzberg S. L. (2012). Fast gapped-read alignment with Bowtie 2. Nat. Method 9, 357–359. doi: 10.1038/nmeth.1923 PMC3322381 22388286

[B60] Li Z. Baniaga A. E. Sessa E. B. Scascitelli M. Graham S. W. Rieseberg L. H.. (2015). Early genome duplications in conifers and other seed plants. Sci. Adv. 1, e1501084. doi: 10.1126/sciadv.1501084 26702445 PMC4681332

[B58] Li B. Dewey C. N. (2011). RSEM: accurate transcript quantification from RNA-Seq data with or without a reference genome. BMC Bioinf. 12, 323. doi: 10.1186/1471-2105-12-323 PMC3163565 21816040

[B59] Li C. Wickell D. Kuo L. Y. Chen X. Nie B. Liao X.. (2023). Extraordinary preservation of gene collinearity over three hundred million years revealed in homosporous lycophytes. bioRxiv. doi: 10.1101/2023.07.24.548637v1 PMC10823260 38236735

[B61] Li Z. McKibben M. T. W. Finch G. S. Blischak P. D. Sutherland B. L. Barker M. S. (2021). Patterns and processes of diploidization in land plants. Annu. Rev. Plant Biol. 72, 387–410. doi: 10.1146/annurev-arplant-050718-100344 33684297

[B62] Lynch M. Conery J. S. (2000). The Evolutionary fate and consequences of duplicate genes. Science 290, 1151–1155. doi: 10.1126/science.290.5494.1151 11073452

[B63] Mandáková T. Lysak M. A. (2018). Post-polyploid diploidization and diversification through dysploid changes. Curr. Opin. Plant Biol. 42, 55–65. doi: 10.1016/j.pbi.2018.03.001 29567623

[B64] Marchant D. B. Chen G. Cai S. Chen F. Schafran P. Jenkins J.. (2022). Dynamic genome evolution in a model fern. Nat. Plants 8, 1038–1051. doi: 10.1038/s41477-022-01226-7 36050461 PMC9477723

[B65] Masuyama S. (1979). Reproductive biology of the fern *Phegopteris decursive-pinnata* I. The dissimilar mating systems of diploids and tetraploids. Bot. Mag. Tokyo 92, 275–289. doi: 10.1007/BF02506251

[B66] Masuyama S. (1986). Reproductive biology of the fern *Phegopteris decursive-pinnata* II. Genetic analyses of self-sterility in diploids. Bot. Mag. Tokyo 99, 107–121. doi: 10.1007/BF02488626

[B67] McClintock B. (1984). The Significance of responses of the genome to challenge. Science 226, 792–801. doi: 10.1126/science.15739260 15739260

[B68] Nakato N. Masuyama S. (2021). Polyploid progeny from triploid hybrids of *Phegopteris decursivepinnata* (Thelypteridaceae). J. Plant Res. 134, 195–208. doi: 10.1007/s10265-021-01255-x 33559786

[B69] Nakato N. Ootsuki R. Murakami N. Masuyama S. (2012). Two types of partial fertility in a diploid population of the fern *Thelypteris decursive-pinnata* (Thelypteridaceae). J. Plant Res. 125, 465–474. doi: 10.1007/s10265-011-0461-7 22038490

[B70] Orr H. A. (1996). Dobzhansky, Bateson, and the genetics of speciation. Genetics 144, 1331–1335. doi: 10.1093/genetics/144.4.1331 8978022 PMC1207686

[B71] Pelosi J. A. Kim E. H. Barbazuk W. B. Sessa E. B. (2022). Phylotranscriptomics illuminates the placement of whole genome duplications and gene retention in ferns. Front. Plant Sci. 13. doi: 10.3389/fpls.2022.882441 PMC9330400 35909764

[B73] Rapp R. A. Udall J. A. Wendel J. F. (2009). Genomic expression dominance in allopolyploids. BMC Biol. 7, 18. doi: 10.1186/1741-7007-7-18 19409075 PMC2684529

[B72] R Core Team (2022). R: A language and environment for statistical computing (Vienna: R Foundation for Statistical Computing). Available at: https://www.R-project.org.

[B74] Rigal M. Becker C. Pélissier T. Pogorelcnik R. Devos J. Ikeda Y.. (2016). Epigenome confrontation triggers immediate reprogramming of DNA methylation and transposon silencing in *Arabidopsis thaliana* F_1_ epihybrids. Proc. Natl. Acad. Sci. U.S.A 113, 2083–2092. doi: 10.1073/pnas.1600672113 PMC4833259 27001853

[B75] Roberts A. Pachter L. (2013). Streaming fragment assignment for real-time analysis of sequencing experiments. Nat. Methods 10, 71–73. doi: 10.1038/nmeth.2251 23160280 PMC3880119

[B76] Robinson M. D. McCarthy D. J. Smyth G. K. (2010). edgeR : a Bioconductor package for differential expression analysis of digital gene expression data. Bioinformatics 26, 139–140. doi: 10.1093/bioinformatics/btp616 19910308 PMC2796818

[B77] Rothfels C. J. Johnson A. K. Hovenkamp P. H. Swofford D. L. Roskam H. C. Fraser-Jenkins C. R.. (2015). Natural hybridization between genera that diverged from each other approximately 60 million years ago. Am. Nat. 185, 433–442. doi: 10.1086/679662 25674696

[B79] Schneider H. Liu H. Clark J. Hidalgo O. Pellicer J. Zhang S.. (2015). Are the genomes of royal ferns really frozen in time? Evidence for coinciding genome stability and limited evolvability in the royal ferns. New Phytol. 207, 10–13. doi: 10.1111/nph.13330 25655176

[B80] Sehrish T. Symonds V. V. Soltis D. E. Soltis P. S. Tate J. A. (2015). Cytonuclear coordination is not immediate upon allopolyploid formation in *Tragopogon miscellus* (Asteraceae) allopolyploids. PloS One 10, e0144339. doi: 10.1371/journal.pone.0144339 26646761 PMC4673006

[B78] Sessa E. B. Der J. P. (2016). Evolutionary genomics of ferns and lycophytes. Adv. Bot. Res. 78, 215–254. doi: 10.1016/bs.abr.2016.02.001

[B81] Shan S. Boatwright J. L. Liu X. Chanderbali A. S. Fu C. Soltis P. S.. (2020). Transcriptome dynamics of the inflorescence in reciprocally formed allopolyploid *Tragopogon miscellus* (Asteraceae). Front. Genet. 11. doi: 10.3389/fgene.2020.00888 PMC7423994 32849847

[B82] Sharbrough J. Conover J. L. Tate J. A. Wendel J. F. Sloan D. B. (2017). Cytonuclear responses to genome doubling. Am. J. Bot. 104, 1277–1280. doi: 10.3732/ajb.1700293 29885242

[B83] Shimizu K. K. (2022). Robustness and the generalist niche of polyploid species: Genome shock or gradual evolution? Curr. Opin. Plant Biol. 69, 102292. doi: 10.1016/j.pbi.2022.102292 36063635

[B84] Shimizu-Inatsugi R. Terada A. Hirose K. Kudoh H. Sese J. Shimizu K. K. (2016). Plant adaptive radiation mediated by polyploid plasticity in transcriptomes. Mol. Ecol. 26, 193–207. doi: 10.1111/mec.13738 27352992

[B85] Sigel E. M. Der J. P. Windham M. D. Pryer K. M. (2019). Expression level dominance and homeolog expression bias in recurrent origins of the allopolyploid fern *Polypodium hesperium* . Am. Fern J. 109, 224. doi: 10.1640/0002-8444-109.3.224

[B86] Sigel E. M. Windham M. D. Pryer K. M. (2014). Evidence for reciprocal origins in *Polypodium hesperium* (Polypodiaceae): A fern model system for investigating how multiple origins shape allopolyploid genomes. Am. J. Bot. 101, 1476–1485. doi: 10.3732/ajb.1400190 25253708

[B87] Šímová I. Herben T. (2012). Geometrical constraints in the scaling relationships between genome size, cell size and cell cycle length in herbaceous plants. Proc. R. Soc B. 279, 867–875. doi: 10.1098/rspb.2011.1284 PMC3259922 21881135

[B88] Song Q. Chen Z. J. (2015). Epigenetic and developmental regulation in plant polyploids. Curr. Opin. Plant Biol 24, 101–109. doi: 10.1016/j.pbi.2015.02.007 25765928 PMC4395545

[B90] Subrahmanian N. Remacle C. Hamel P. P. (2016). Plant mitochondrial complex I composition and assembly: A review. Biochim. Biophys. Acta - Biomembr. 1857, 1001–1014. doi: 10.1016/j.bbabio.2016.01.009 26801215

[B89] Szövényi P. Gunadi A. Li F. W. (2021). Charting the genomic landscape of seed-free plants. Nat. Plants 7, 554–565. doi: 10.1038/s41477-021-00888-z 33820965

[B91] Van De Peer Y. Mizrachi E. Marchal K. (2017). The evolutionary significance of polyploidy. Nat. Rev. Genet. 18, 411–424. doi: 10.1038/nrg.2017.26 28502977

[B92] Vasudevan A. Lévesque-Lemay M. Edwards T. Cloutier S. (2023). Global transcriptome analysis of allopolyploidization reveals large-scale repression of the D-subgenome in synthetic hexaploid wheat. Commun. Biol. 6, 426. doi: 10.1038/s42003-023-04781-7 37069312 PMC10110605

[B93] Wang X. Morton J. A. Pellicer J. Leitch I. J. Leitch A. R. (2021). Genome downsizing after polyploidy: mechanisms, rates and selection pressures. Plant J. 107, 1003–1015. doi: 10.1111/tpj.15363 34077584

[B94] Watano Y. Masuyama S. (1991). Inbreeding in natural populations of the annual polyploid fern *Ceratopteris thalictroides* (Parkeriaceae). Syst. Bot. 16, 705–714. doi: 10.2307/2418872

[B95] Wendel J. F. Lisch D. Hu G. Mason A. S. (2018). The long and short of doubling down: polyploidy, epigenetics, and the temporal dynamics of genome fractionation. Curr. Opin. Genet. Dev. 49, 1–7. doi: 10.1016/j.gde.2018.01.004 29438956

[B96] Whitney K. D. Ahern J. R. Campbell L. G. Albert L. P. King M. S. (2010). Patterns of hybridization in plants. Plant Ecol. Evol. Syst. 12, 175–182. doi: 10.1016/j.ppees.2010.02.002

[B97] Wood T. E. Takebayashi N. Barker M. S. Mayrose I. Greenspoon P. B. Rieseberg L. H. (2009). The frequency of polyploid speciation in vascular plants. Proc. Natl. Acad. Sci. U.S.A. 106, 13875–13879. doi: 10.1073/pnas.0811575106 19667210 PMC2728988

[B99] Wu J. Lin L. Xu M. Chen P. Liu D. Sun Q.. (2018). Homoeolog expression bias and expression level dominance in resynthesized allopolyploid *Brassica napus* . BMC Genomics 19, 586. doi: 10.1186/s12864-018-4966-5 30081834 PMC6080508

[B98] Wu H. Yu Q. Ran J.-H. Wang X.-Q. (2021). Unbiased subgenome evolution in allotetraploid species of *Ephedra* and its implications for the evolution of large genomes in gymnosperms. Genome Biol. Evol. 13, evaa236. doi: 10.1093/gbe/evaa236 33196777 PMC7900875

[B102] Xia Z. Q. Wei Z. Y. Shen H. Shu J. P. Wang T. Gu Y. F.. (2022). Lycophyte transcriptomes reveal two whole-genome duplications in Lycopodiaceae: Insights into the polyploidization of *Phlegmariurus* . Plant Divers. 44, 262–270. doi: 10.1016/j.pld.2021.08.004 35769590 PMC9209867

[B100] Yoo M.-J. Liu X. Pires J. C. Soltis P. S. Soltis D. E. (2014). Nonadditive gene expression in polyploids. Annu. Rev. Genet. 48, 485–517. doi: 10.1146/annurev-genet-120213-092159 25421600

[B101] Yoo M.-J. Szadkowski E. Wendel J. F. (2013). Homoeolog expression bias and expression level dominance in allopolyploid cotton. Heredity 110, 171–180. doi: 10.1038/hdy.2012.94 23169565 PMC3554454

[B103] Zhong Y. Liu Y. Wu W. Chen J. Sun C. Liu H.. (2022). Genomic insights into genetic diploidization in the homosporous fern *Adiantum nelumboides* . Genome Biol. Evol. 14, evac127. doi: 10.1093/gbe/evac127 35946426 PMC9387920

