# Supplementary material for: Subgenome evolutionary dynamics in allotetraploid ferns: insights from the gene expression patterns in the allotetraploid species Phegopteris decursivepinnata (Thelypteridaceae, Polypodiales)
Source: Front Plant Sci. 2024 Jan 9;14:1286320. doi: 10.3389/fpls.2023.1286320 (PMC10803465; doi:10.3389/fpls.2023.1286320)
Supplement: Supplementary file 1 [file DataSheet_1.zip › Data Sheet.docx]

Supplementary Material

# Supplementary Figures and Tables

## Supplementary Figures

**Supplementary Figure 1. Plastid and nuclear gene phylogenies of *Phegopteris decursivepinnat*a complex including artificial F_1_ hybrids.** OTUs labeled as ‘A’ and ‘B’ in each phylogeny indicate haplotype or allele derived from *P. koreana* and *P. taiwaniana,* respectively (see Fujiwara et al. 2021). Pink and blue color in the hybrids show hybrid with *P. koreana* as maternal lineage and with *P. taiwaniana* as maternal lineage, respectively.

**
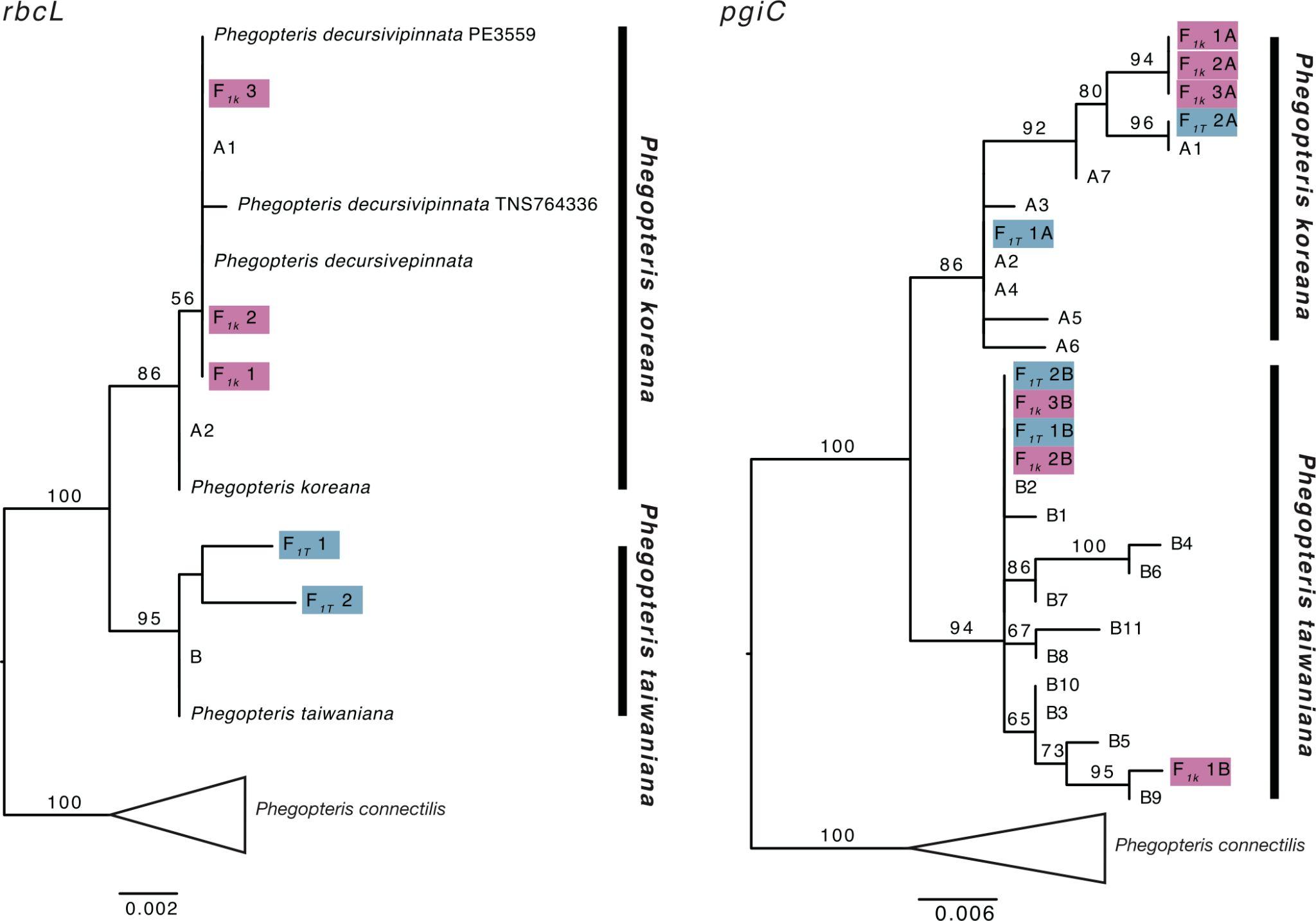
**

**Supplementary Figure 2. Venn diagram of co-expressed orthologous genes among five groups including *P. koreana*, *P. taiwaniana*, F_1K_ hybrid, F_1T_ hybrid and allotetraploid, *P. decursivepinnata* for orthogroups with up to 1 gene of each species (Supplementary Table S3).** Blue-shaded part is single-copy orthologous genes shared between the two parental species, which used as mapping reference.

**
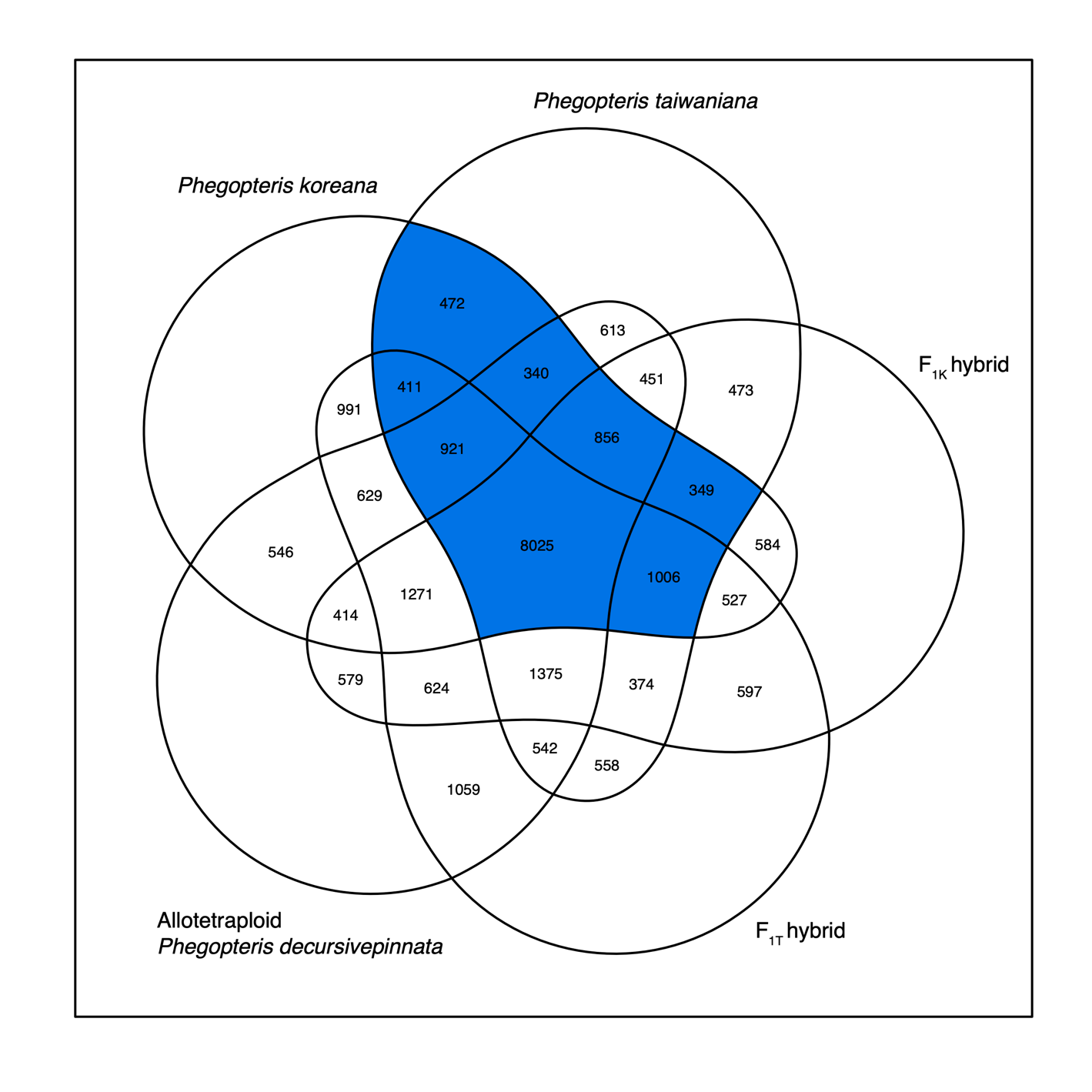
**

**Supplementary Figure 3. MDS plots of expression matrix in the dataset with different TPM filtering, TPM >0, TPM >0.5, and TPM > 1.** Each plot contains all replicates of the parental species, *P. koreana* (K1-3) and *P. taiwaniana* (T1-3), and each subgenome of all replicates of F_1K_ hybrid (F_1K__1-3), F_1T_ hybrid (F_1T__1-2), and allotetraploid (D1-3). The year after the sample name indicate the year the sample was collected.


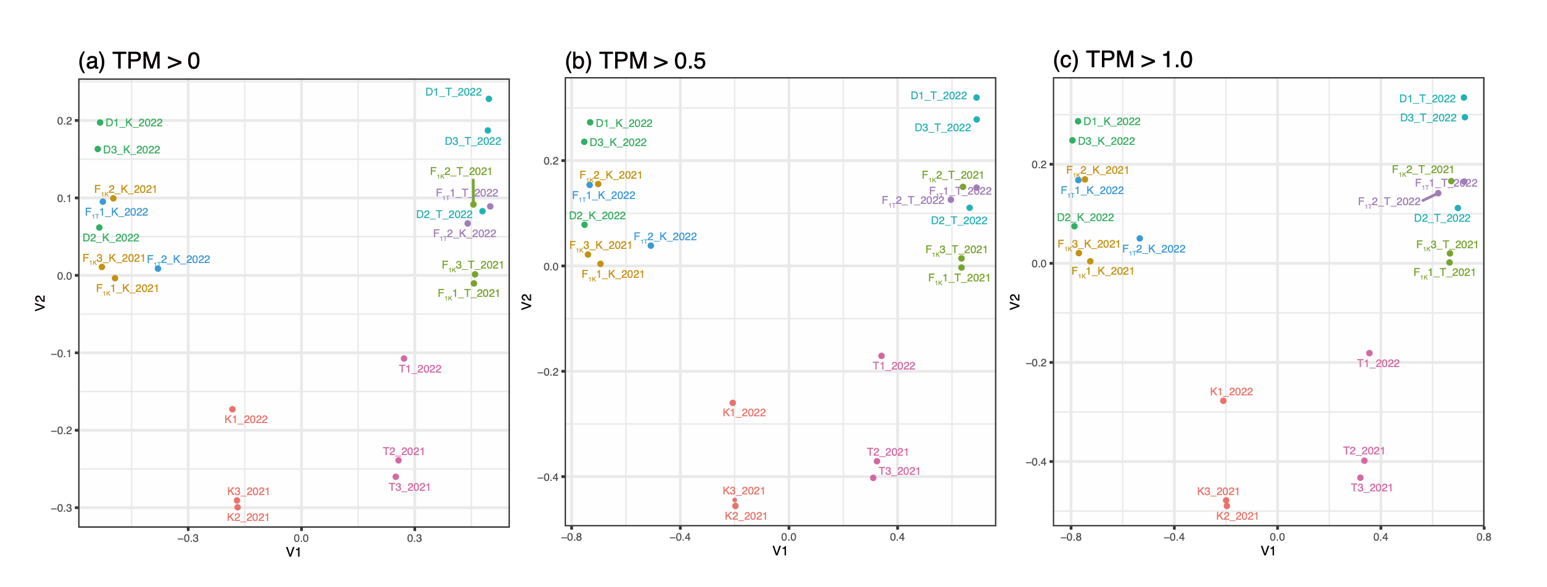


**Supplementary Figure 4. Twelve categories for differential expression states in the synthesized F1 hybrids (F_1K_ and F_1T_) and the natural allotetraploids relative to the diploid parents, in the datasets with TPM > 0.0 (a) and TPM > 1.0 (b).** Roman numerals indicate categories as described by Rapp et al. (2009). Numbers show the number of genes assigned to each category and the rates are shown in parentheses. Schematic figures show the gene expression levels relative to the parents (K: *P. korean*a, P: F_1K_, F_1T_, or *P. decursivepinnata*, T: *P. taiwaniana)*.

**
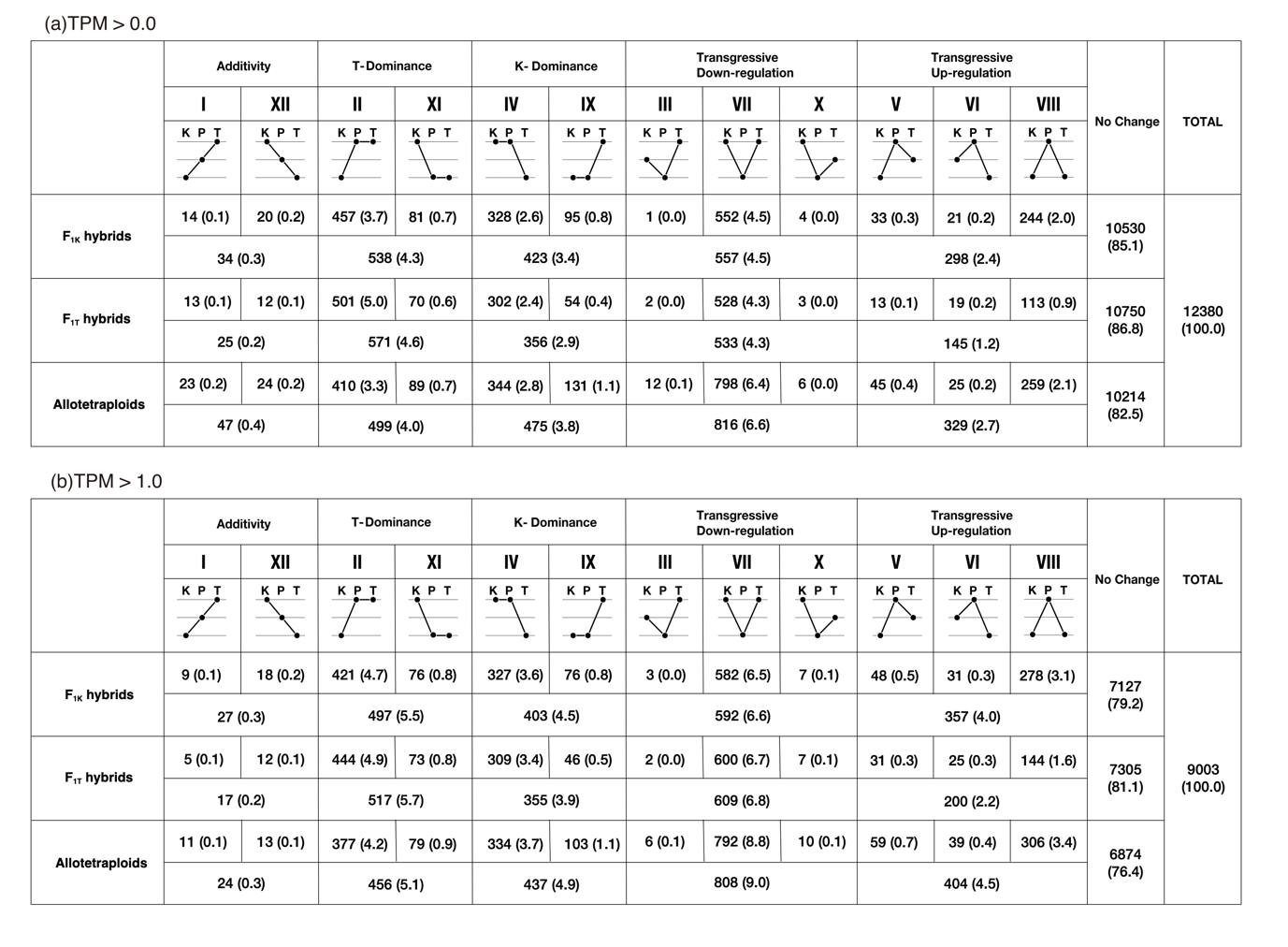
**

**Supplementary Figure 5. Diagram showing categorical changes in the differential expression of genes from the synthesized F_1K_ hybrids to the allotetraploids, in the datasets with TPM > 0.0 and TPM > 1.0.** NC, KD, TD, ADD, TUR and TDR are the abbreviations of “No Change”, “K- dominance”, “T- dominance”, “Additivity”, “Transgressive Up-Regulation”, and “Transgressive Down-Regulation” respectively. Numbers with arrows indicate the number of genes whose categories changed from *F_1K_* to *P. decursivepinnata*.


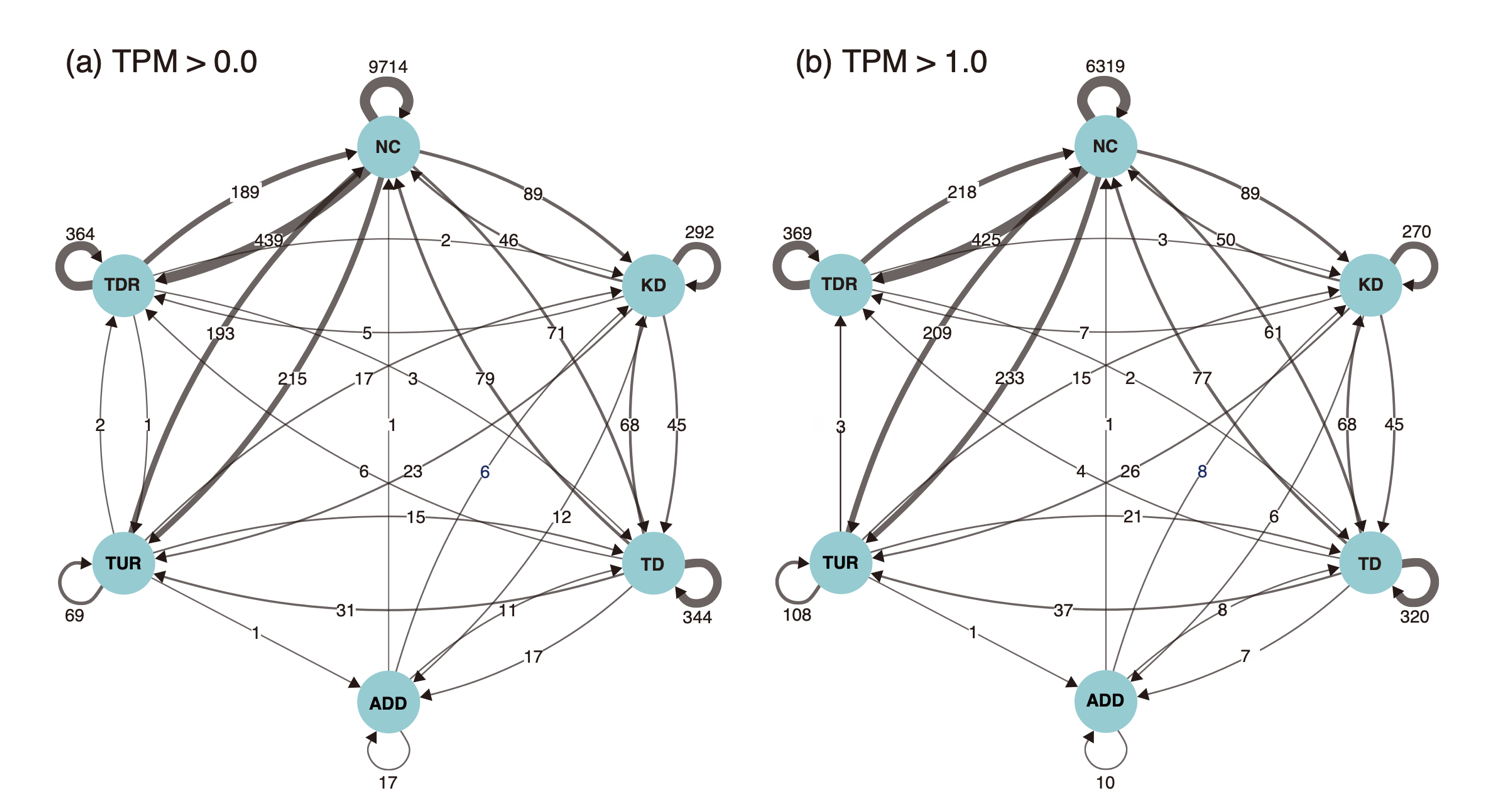


**Supplementary Figure 6.** **Patterns in the expression level ratio between homoeologs derived from *P. koreana* and *P. taiwaniana* (sub)genomes, in the dataset with TPM > 0.0 (a) and TPM > 1.0 (b).** Violin plots showing the log_2_(fold change (FC)) between the orthologous genes of the parental species (*P. koreana* and *P. taiwaniana*), the parental alleles of the F_1K_ and F_1T_ hybrids, and the homoeologous genes of *P. decursivepinnanta*. The center line indicates the median, and the box limits represent the interquartile range. The whiskers represent the largest and smallest values within 1.5 times the interquartile range above and below the 75th and 25th percentiles, respectively.

**
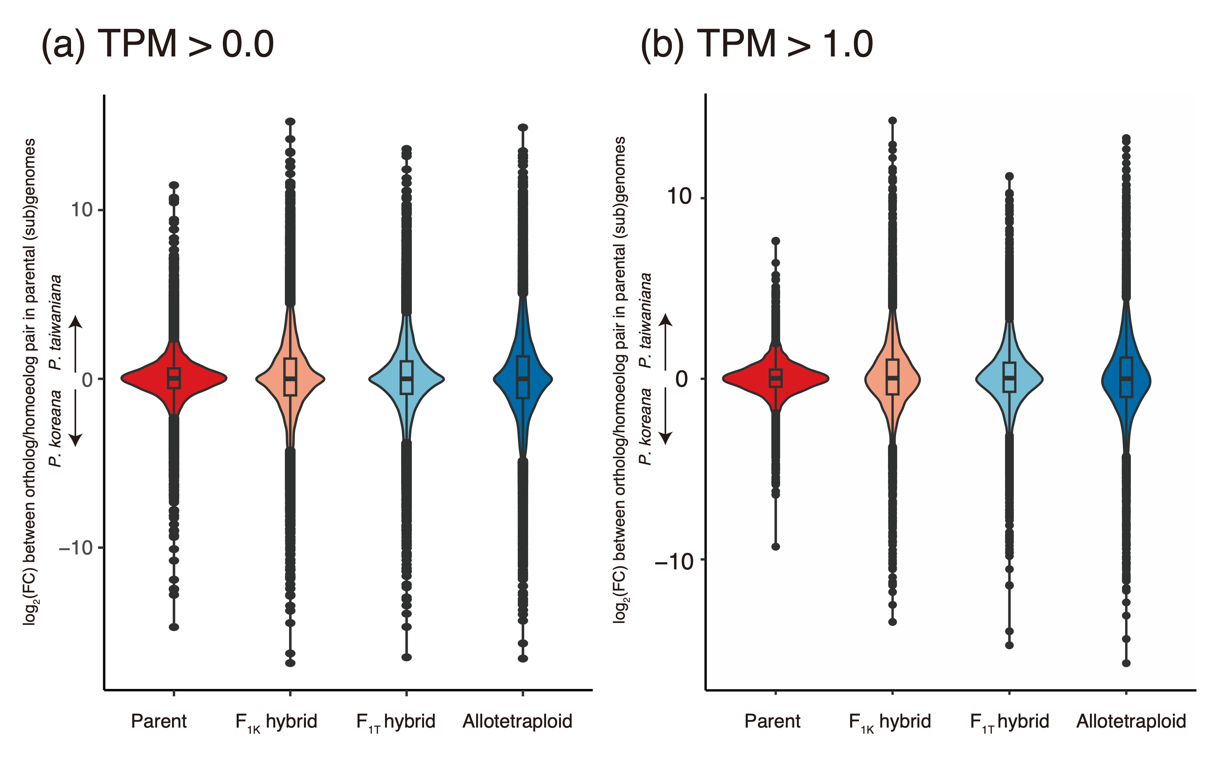
**

**Supplementary Figure 7. Homoeolog expression bias (HEB) in the F_1K_ hybrids and the allotetraploids in the dataset with TPM > 0.0 (a, b and c), and TPM > 1.0 (e, f, g).** (a, e) Synthesized F_1K_ hybrids, (b,f) Synthesized F_1T_ hybrids. (c,g) Allotetraploid, *P. decursivepinnata*. Histograms showing the “magnitude” of HEB calculated from the formula; log_2_((F_1K_T-homoeolog_ /F_1K_K-homoeolog_) / (Pt__ortholog_ /Pk__ortholog_)) (a, e), log_2_((F_1T_T-homoeolog_ /F_1T_K-homoeolog_) / (Pt__ortholog_ /Pk__ortholog_)) (b, f), and log_2_((Pd__T-homoeolog_ /Pd__K-homoeolog_)/log_2_(Pt__ortholog_ /Pk__ortholog_)) (c, g). Blue and orange bars indicate T- and K-bias gene pairs, respectively.


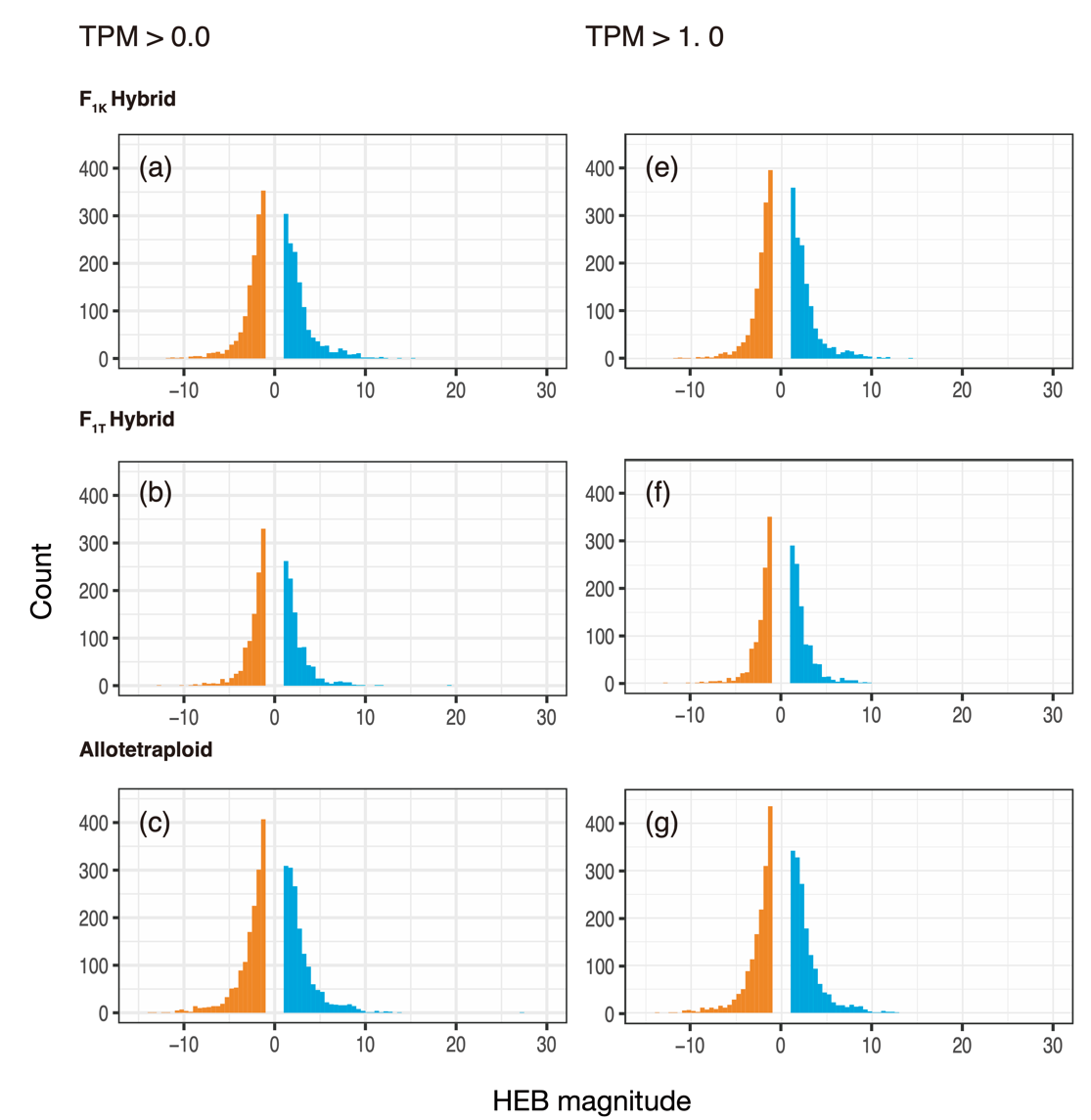


**1.2. Supplementary Tables**

**Supplementary Table 1.** **Voucher information for samples collected in this study.**

**Supplementary Table 2. Information for samples used for the sporophyte production of the parental species and artificial F_1_ hybrid production.**

**Supplementary Table 3. Summary for sequencing, assembly and filtering in this study.**

**Supplementary Table 4. The list of strong K-biased genes (“magnitude” <-10) in *P. decursivepinnata*.**

**Supplementary Table 5. The list of strong T-biased genes (“magnitude” <-10) in *P. decursivepinnata*.**

**Supplementary Table 6. The list of strong K-biased genes (“magnitude” <-10) in F_1K_.**

**Supplementary Table 7. The list of strong T-biased genes (“magnitude” <-10) in F_1K_.**
